# The Role of ncRNAs in Cardiac Infarction and Regeneration

**DOI:** 10.3390/jcdd10030123

**Published:** 2023-03-15

**Authors:** Sheila Caño-Carrillo, Estefanía Lozano-Velasco, Juan Manuel Castillo-Casas, Cristina Sánchez-Fernández, Diego Franco

**Affiliations:** 1 Cardiovascular Development Group, Department of Experimental Biology, University of Jaen, 23071 Jaen, Spain; 2 Medina Foundation, 18007 Granada, Spain

**Keywords:** cardiac infarction, regeneration, miRNAs, lncRNAs, circRNAs

## Abstract

Myocardial infarction is the most prevalent cardiovascular disease worldwide, and it is defined as cardiomyocyte cell death due to a lack of oxygen supply. Such a temporary absence of oxygen supply, or ischemia, leads to extensive cardiomyocyte cell death in the affected myocardium. Notably, reactive oxygen species are generated during the reperfusion process, driving a novel wave of cell death. Consequently, the inflammatory process starts, followed by fibrotic scar formation. Limiting inflammation and resolving the fibrotic scar are essential biological processes with respect to providing a favorable environment for cardiac regeneration that is only achieved in a limited number of species. Distinct inductive signals and transcriptional regulatory factors are key components that modulate cardiac injury and regeneration. Over the last decade, the impact of non-coding RNAs has begun to be addressed in many cellular and pathological processes including myocardial infarction and regeneration. Herein, we provide a state-of-the-art review of the current functional role of diverse non-coding RNAs, particularly microRNAs (miRNAs), long non-coding RNAs (lncRNAs), and circular RNAs (circRNAs), in different biological processes involved in cardiac injury as well as in distinct experimental models of cardiac regeneration.

## 1. Introduction

Myocardial infarction (MI) represents the most prevalent cardiovascular disease worldwide [1]. From a pathological perspective, MI is defined as cardiomyocyte (CM) cell death due to lack of oxygen supply, i.e., an ischemic event [2]. Ischemia leads to the necrosis and cell death of the affected myocardium. Moreover, during the reperfusion process, the sudden influx of oxygenated blood drives the generation of reactive oxygen species (ROS), promoting oxidative stress and an extra wave of cell death [3]. As it is well known, the adult human heart has low levels of cardiomyocyte proliferation, thereby limiting its healing capacity. However, it must be considered that if the ischemic process is shorter than 20 min, CMs can survive after the restoration of coronary flow, while longer ischemic periods facilitate a process wherein millions of CMs lose their contraction potential and die. These events promote pro-inflammatory scenarios within the infarcted area and cardiac fibroblasts (CFs) become activated, generating a fibrotic scar. More concretely, when MI occurs and the necrosis process has started, an inflammatory response is prompted by the CMs death. Pointedly, in the first wave of heart healing, necrotic cells must be cleared away by the proinflammatory extracellular-matrix (ECM)-degrading component; this process is known as efferocytosis [4]. Afterwards, inflammation is deactivated, and ECM deposition is activated to form the new scar [4]. These two processes are well coordinated and involve fine-tuned cross-talk among different cell types, such as cardiac myocytes, neutrophils, macrophages, fibroblasts, endothelial cells, and nerve cells, among which macrophages and fibroblasts are the two cell populations with the most important roles during the post-MI response. Finally, inflammation and scar formation lead to a loss of contractile function, which eventually induces heart failure (HF) [3,5].

## 2. The Process of Inflammation during Cardiac Injury

In injured cardiac cells, an inflammatory process is triggered as a consequence of the activation of the Toll receptor (TLR) and/or nuclear factor-κB (NF-κB signaling pathways through the modulation of SOCS3 (suppressor of cytokine signaling 3) [6,7,8]. In the inflammatory phase, necrotic CMs release danger-associated molecular patterns (DAMPs). These molecules bind to their receptors in the surviving parenchymal cells and infiltrating leukocytes, thereby triggering the delivery of inflammatory actors, i.e., inflammatory cytokines such as CXC chemokines for neutrophil chemoattraction, CC chemokines that attract monocytes and T-lymphocytes, cell adhesion molecules, and complement factor B [4]. The expression of the pro-inflammatory genes is driven by DAMP–receptor binding, which induces an intracellular signaling pathway that leads to the activation of mitogen-activated protein kinases (MAPKs) and NF-κB [4].

At the cellular level, CMs, immune cells, vascular cells, and fibroblasts are key actors in the inflammatory response, although their precise roles remain largely unknown. Necrotic CMs are responsible for DAMPs’ release in the infarcted area, while the activation of endothelial cells is needed for the extravasation of leukocytes as neutrophils [9]. Emigrated neutrophils become the predominant cell type in the infarct zone within the first 24 h after MI and aid in the clearance of dead cells and matrix debris from the infarct zone. After 7 days post-MI, neutrophil levels decline due to the reduction in neutrophil extravasation by an anti-inflammatory process; however, the fibrotic response is enhanced, promoting HF [4,10]. Monocytes are immune cells that can differentiate into macrophages [11], and phagocytosis is one of their principal functions [12]. Resident macrophages are a heterogeneous population within the adult heart [13]. Like neutrophils, macrophages have a polarized status depending on their function. M1 macrophages play a pro-inflammatory role on day 1 post-MI, with phagocytic and proteolytic functions. Later, M1 macrophages change into M2 macrophages, which perform an anti-inflammatory and reparative role around day 7 post-MI due to the release of cytokines [10,14,15,16,17]. It is essential to highlight that M1 macrophages induce a positive effect by producing pro-inflammatory exosomes (M1-exos) that accelerate injury repair after MI and promote angiogenesis [18]. However, if such M1 macrophages’ effect is maintained over time, it can trigger ECM degradation [19], thus decreasing the capacity for regeneration [20,21].

Concerning CFs, it is important to highlight that they represent an abundant population of cells in the adult mammalian myocardium that maintain homeostatic ECM conditions. However, after the release of DAMPs, CFs become activated, secreting cytokines and chemokines that may prevent fibrotic scar formation before cell death, and matrix debris are eliminated from the infarct zone [4]. Furthermore, several studies have demonstrated tight cross-talk among macrophages, neutrophils, fibroblasts, and endothelial cells [10]. Finally, during the inflammation process, the ECM plays an essential role as a structural scaffold and in the transduction of molecular signals, wherein it induces cytokine and chemokine segregation by endothelial and immune cells [4].

## 3. The Process of Fibrosis during Cardiac Injury

As mentioned before, the infarct zone is cleared of dead cells and matrix debris during the inflammation process. This step is followed by a proliferative phase that leads to cardiac repair, in which anti-inflammatory pathways are activated and myofibroblasts and vascular cells infiltrate the wounded area. It is necessary to suppress the inflammatory process for cardiac repair because a lack of optimal preservation of the cardiac structure could entail worse effects at the functional level [5]. During the clearance of dead cells, anti-inflammatory cytokines are released; concomitantly, infiltrated neutrophils undergo apoptosis [5]. After the inflammatory response, CFs are the main cell type within the infarcted area, and a phenotypic change occurs, where CFs transdifferentiate into myofibroblasts. These cells, which undergo increased synthesis of both structural and matricellular ECM proteins, present a proliferative phenotype and high expression of contractile proteins, such as α-smooth muscle actin (SMA) [22]. This transdifferentiation process is mediated by the activation of the transforming growth factor β (TGF-β), the deposition of fibronectin and other matrix proteins, and the removal of the pro-inflammatory inductor IL-1β [22].

## 4. Inductive and Transcriptional Regulatory Mechanisms Driving Cardiac Injury and Regeneration

Transcriptional regulation and its associated regulatory mechanisms are key components of cardiac regeneration. Several laboratories have deciphered the role of different transcription and growth factors that have the ability to maintain, in neonatal mice, or diminish, in adult mice, cardiac regenerative capacity [23]. A few years ago, Porrello’s lab analyzed the different transcriptional networks as well as signaling pathways and cellular processes in adult and neonatal injured hearts [24]. They found that neonatal CMs were enriched in transcription factors related to the cell cycle, such as E2f1 and Foxm1. In contrast, adult CMs were enriched in transcription factors related to autophagy, such as Tfeb (transcription factor EB), Amfr (autocrine motility factor receptor), Gabarap (GABA-type-A-receptor-associated protein), and oxidative stress-related genes such as Sod1 (superoxide dismutase 1) [24].

Interestingly, follistatin-like 1 (Fstl1) is expressed in the normal epicardium in mice, although its expression is reduced after cardiac injury is triggered in the myocardium. It has been evidenced that Fstl1 derived from epicardial cells promotes the proliferation of CMs. However, Fslt1 from the myocardium have lost this ability, probably due to post-translational modifications [25]. In the same line, Neuregulin1 (Nrg1) modulates CM proliferation in mammals, and its administration improves cardiac regeneration. This process seems to be mediated by an NRG1 receptor, namely, Erbb2 (erb-b2 receptor tyrosine kinase 2), whose expression dramatically diminishes in neonatal mice one week after birth [26]. In contrast, Erbb2 levels in adult zebrafish are maintained, thereby contributing to the preservation of cardiac regeneration via the modulation of Nrg1 levels [26]. Moreover, it must be considered that chromatin remodeling is associated with the inability of adult CMs to recapitulate neonatal proliferative programs. In neonatal injured CMs, a euchromatic state was found within genomic regions related to the cell cycle and inflammation genes; however, these euchromatic regions become more condensed in postnatal and adult injured CMs [24]. Overall, these studies provide an initial demonstration of the important contributions of inductive and transcriptional regulatory mechanisms during cardiac regeneration.

## 5. The Emerging Impact of Non-Coding RNAs’ Regulation of Cardiac Injury and Regeneration

The molecular bases that govern cardiac regeneration are very complex, including both coding RNAs and non-coding RNAs (ncRNAs) as pivotal modulators. Researchers began addressing the impacts of non-coding RNAs some years ago, and it was through such research that it was discovered that they are powerful regulators of a multitude of cellular and pathological processes such as MI, hypertrophy, HF, and arrhythmias [27]. ncRNAs are functional RNA molecules (without protein-coding functions) that play an essential role in distinct biological and physiological processes as well as pathological disorders [28]. According to the number of nucleotides contained and their characteristics [28], ncRNAs are classified into (i) small non-coding RNAs (≤200 nucleotides), including microRNAs (miRNAs), small nucleolar RNAs (snoRNAs), piwi-interacting RNAs (piRNAs), and transfer RNA (tRNAs); (ii) long non-coding RNAs (>200 nucleotides), including intronic, enhancer, circular, and intergenic lncRNAs; and (iii) circular RNA (circRNA), which lacks free ends and comprises a wide range of ncRNAs. This third emerging class is produced by a non-canonical splicing event (back-splicing), a process in which a downstream splice-donor site is covalently linked to an upstream splice-acceptor site. The canonical function of miRNAs is to modulate gene expression at the post-transcriptional level by recognizing and binding to target mRNAs and triggering their degradation [29,30]. Several studies have demonstrated that miRNAs are closely related to inflammation, fibrosis, and angiogenesis after MI [31,32]. The molecular mechanisms of long non-coding RNAs (lncRNA) are more complex than those of miRNAs because lncRNAs can exert their functions both at the transcriptional and post-transcriptional levels, interacting with all types of RNA molecules, proteins, and different chromatic modulators [33]. The participation of lncRNAs in MI has been recently evidenced. The genome-wide profiling of the cardiac transcriptome after MI has evidenced deregulated heart-specific lncRNAs [34]. Finally, circRNAs act by binding to proteins or miRNAs, interfering with them and blocking their function [35]. Evidence has demonstrated that circRNAs’ expression is impaired after MI [36]. Scientists from different countries found that circulating ncRNAs are sensitive biomarkers for cancer and other kinds of disease, including MI. These findings indicate that the identified biomarkers for MI offer great potential for clinical applications [37].

Within the MI context, the ability to link ncRNAs with the main cardiac gene-regulatory networks that drive the main biological processes activated after injury, such as inflammation and fibrosis, may provide a new opportunity for therapeutic intervention via regenerative medicine applied to the heart. The generation of new knowledge about this pathological process has become the primary concern within the field of cardiovascular research due to the high socioeconomic burden of acute MI and its chronic consequences in surviving patients. The current manuscript provides a comprehensive review of the roles of ncRNAs (miRNAs, lncRNAs, and circRNAs) during ischemic myocardial injury, in post-infarction remodeling, and in cardiac repair mechanisms.

### 5.1. The Functional Roles of miRNAs in the Cardiac Inflammatory Response

In recent years, an increasing number of miRNAs have been reported to play distinct roles during the inflammatory response in MI. Among these miRNAs, six, namely, miR-181, miR-155, miR-21, miR-146, miR-133, and miR-130, have been considerably well studied in multiple reports, as detailed below, including through in vitro and in vivo models. Importantly, the functional roles of these miRNAs can be exerted, via their secretion into extracellular vesicles, in remote cells, wherein they directly modulate transcription factors and/or growth factors in the targeted cells. In addition, their function can be mediated by their interaction with other non-coding RNA molecules such as lncRNAs and circRNAs. In the following paragraphs, we will summarize the current state-of-the-art knowledge of those miRNAs involved in cardiac inflammatory processes during cardiac injury.

#### 5.1.1. The Role of miR-181

**Figure 1:**
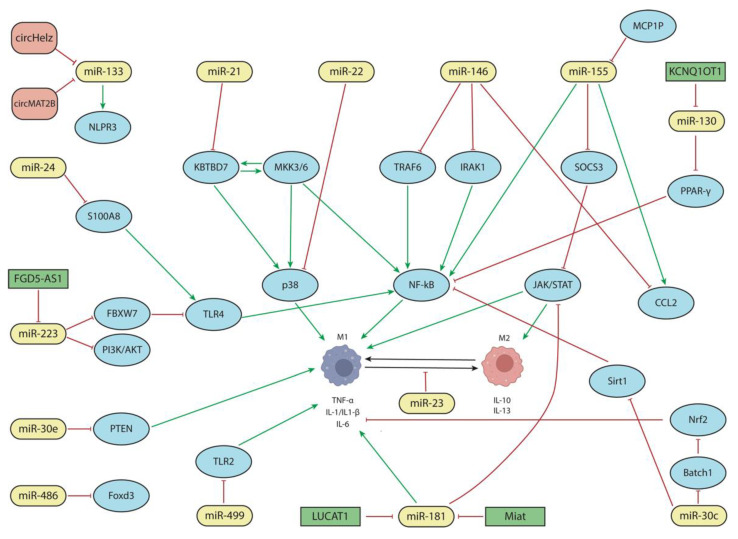
Cardiac inflammation. Schematic representation of the main miRNA, lncRNA, and circRNA gene-regulatory networks reported to play a role in distinct signaling pathways leading to cardiac inflammation. Green arrows delineate the ncRNAs that activate the signaling pathway while those depicted in red inhibit such signaling pathways. Note that many ncRNAs preferentially modulate NF-κB signaling, thus influencing M1 to M2 macrophage activation. circRNAs are depicted in green, microRNAs are depicted in yellow, and the different signaling pathways modulating the inflammatory response are depicted in blue.

As previously stated, the functional role of miR-181 in cardiac inflammation is particularly well documented. Several in vitro and in vivo studies have demonstrated that high levels of miR-181 lead to the inhibition of myocardial inflammation via the modulation of pro-inflammatory markers (such as tumor necrosis factor-α (TNF-α) and IL-1β) and oxidative stress; through the regulation of ROS components such as SOD1 (superoxide dismutase 1) and SOD2; and by targeting ATF2 (activating transcription factor 2) (Figure 1) [38,39]. Moreover, LUCAT1 and MIAT lncRNAs are regulators of miR-181a-5p, thereby modulating inflammation (TNF-α, IL6, and IL1β), oxidative stress (SOD levels), apoptosis, and cell viability in vitro, which was demonstrated in an in vitro model of CM injury or ischemia–reperfusion, as well as in vivo by modulating the JAK/STAT signaling pathway (Figure 1) [40,41].

#### 5.1.2. The Role of miR-155

miR-155 is also one of the main non-coding RNAs involved in the inflammatory process occurring after MI [42,43]. miR-155’s biogenesis is regulated by MCPIP (monocyte chemotactic protein-induced protein), which negatively modulates inflammation [44]. MCPIP is not only expressed in CMs but plays an essential role in myocardial cells during inflammation after MI by down-regulating miR-155 and NF-κB signaling (Figure 1) [45]. miR-155 regulates the differentiation and polarization of macrophages through the JAK2/STAT3 signaling pathway, particularly targeting SOC3 (Figure 1) [46,47,48,49,50].

M1-macrophage-produced exosomes are loaded with miR-155 [18,51]. In mice, M2 macrophage polarization is necessary for cardiac regeneration that is mediated by a decrease in miR-155; however, the inflammatory process is maintained due to an increase in miR-155 and the conversion of M2 to M1 mediated by CCL2 (C-C motif chemokine ligand 2) expression (Figure 1) [52,53,54]. A similar scenario is observed in human patients, where miR-155 expression is correlated with the absence (upregulation) or presence (downregulation) of ventricular rupture [18,55,56]. Curiously, while there is ample evidence of the direct involvement of miR-155 in the inflammatory process during cardiac infarction through its pro-inflammatory role, no evidence has been reported on its regulation by distinct non-coding RNAs, such as lncRNAs and/or circRNAs.

#### 5.1.3. The Role of miR-21

Several in vitro studies have demonstrated an anti-inflammatory response regulated by miR-21 [57,58]. In this context, miR-21 overexpression inhibits M1 polarization by reducing ROS production and the levels of some pro-inflammatory markers (IL-6 and TNF-α) while promoting high levels of anti-inflammatory markers (Arg-1 and IL-10) (Figure 1) [59,60]. In vivo analysis has recapitulated the anti-inflammatory and pro-angiogenic roles of miR-21 [61,62]. In an ischemia/reperfusion mouse model, extracellular vesicles (EVs) loaded with CD47 and miR-21 were observed to exert an anti-inflammatory effect by decreasing the expression of IL-1 and IL-6 and increasing the expression of the anti-inflammatory cytokine, IL-13. Moreover, this treatment was also associated with a decrease in the levels of CD68^+^ macrophages, which number among the main markers of M0 macrophage differentiation. Therefore, these EVs reduce the number of macrophages with the capacity to differentiate into M1 or M2 forms (Figure 1) [63,64,65].

The anti-inflammatory action of miR-21 has been demonstrated in miR-21 knock-out mice, in which IL-1, IL-6, and TNF-α are increased as a consequence of the activation of p38 and NF-κB signaling mediated by the interaction of KBTBD7 (Kelch repeat and BTB-domain-containing protein 7) with MKK3/6 (Figure 1) [22,57]. Curiously, miR-21 is elevated in human patients with MI [66].

#### 5.1.4. The Role of miR-146

miR-146 also participates in the inflammatory response after MI, acting as a negative regulator [67]. Several studies have observed high levels of miR-146 after MI that control inflammation by the regulation of NF-κB signaling through the modulation of TNF-receptor-associated factor 6 (TRAF6), thus promoting M1 macrophage polarization (Figure 1) [55,66,68,69,70,71,72,73,74]. miR-146 also downregulates IRAK1 (interleukin-1 receptor-associated kinase 1), thereby decreasing the production of pro-inflammatory cytokines (Figure 1) [75]. Several studies have also reported a relationship between miR-155 and miR-146 [55,76] given the role of miR-146 in dampening excessive inflammation [67]. miR-146 downregulates (while miR-155 upregulates) CCL2 and also inhibits the IRAK1/TRAF6 signaling pathway, thus leading to pro-inflammatory cytokine production (Figure 1) [16,18,77,78,79].

#### 5.1.5. The Role of miR-133

The role of miR-133 has not been directly linked to cardiac inflammation. However, it has been indirectly linked to cardiac inflammation throughout the regulation of this microRNA by distinct circRNAs, i.e., circMAT2b and circHelz (Figure 1). Both circRNAs act in vitro as endogenous sponges of miR-133, reducing its expression. Thus, high levels of these circRNAs in MI decrease cardiac injury [80]. Furthermore, circHelz in vivo essays demonstrated a regulation of the miR-133/NLRP3 (NLR family protein pyrin domain containing 3) axis (Figure 1), leading to low levels of NLRP3 inflammasome that, therefore, decreased myocardial infarct size, pyroptosis, and inflammation and increased cardiac function [81].

#### 5.1.6. The Role of miR-130

In vitro and in vivo experiments have shown that miR-130 targets PPAR-γ (peroxisome proliferator-activated receptor γ). miR-130 downregulates PPAR-γ, increasing the expression levels of proteins associated with fibrosis and inflammation. Therefore, the inhibition of this miRNA confers a cardioprotective effect by inhibiting NFκB-mediated inflammation (Figure 1) [82]. Evidence of miR-130 regulation by lncRNAs has also been reported. For example, lncRNA KCNQ1OT1 plays an important role in MI given that its expression is upregulated in vitro. This lncRNA upregulates the expression of ZNF791 through the miR-130a/ZNF791 axis, exerting an inhibitory effect on this miRNA and thus ameliorating injury in CMs (Figure 1) [83].

In addition to those previously described miRNAs, other miRNAs (miR-24, miR-223, miR-23, miR-22, miR-30, miR-499, and miR-486) have also been reported during cardiac inflammation in different experimental contexts, i.e., MI, ischemia/reperfusion, and HF, yet the current reported evidence is more limited.

#### 5.1.7. The Role of miR-24

In in vivo models of MI, miR-24 improves cardiac function through its negative regulation of S100A8 (S100 calcium-binding protein A8). Due to this inhibition, the arrival of inflammatory cells to the injury zone is reduced by the action of the S100A8/Toll-like receptor 4/MyD-88/NF-κB axis (Figure 1) [84,85,86]. Additionally, miR-24 can also inhibit CCR2 (C-C motif chemokine receptor 2), which is involved in monocyte recruitment [87]. Thus, miR-24 reduces inflammation and fibrosis by inhibiting the S100A8/Toll-like receptor 4/MyD-88/NF-κB and TGF-β pathways, respectively (Figure 1). Furthermore, this miRNA also regulates fibroblast and CM apoptosis, although the mechanism of this process is unclear.

#### 5.1.8. The Role of miR-223

MI also reduces miR-223 levels both in vitro and in vivo, thereby increasing cytokine production (IL-6, IL10, and TNF-α) [88]. This effect is caused by miR-223’s inhibition of FBXW7 (F-box and WD repeat domain containing 7), a key molecule that participates in the inflammatory response regulating the TLR4 (Toll-like receptor 4) signaling pathway (Figure 1) [89,90]. In addition, non-coding RNAs that regulate this miRNA have also been described, for example, lncRNA FGD5-AS1, which negatively regulates miR-223 [91]. Since miR-223 regulates PI3K/AKT, the effect of lncRNA FGD5-AS1 has been related to decreased production of cytokines. Therefore, miR-223 drives an anti-inflammatory situation in MI since low levels of miR-223 increase FBXW7 expression and thus inhibit the TLR4/NF-κB pathway (Figure 1) [92].

#### 5.1.9. The Role of miR-23

miR-23-3p downregulation in acute HF promotes macrophage polarization [93]. In particular, the deletion of 12/15 lipoxygenase (LOX) downregulates miR-23a-3p during acute HF in mice, altering cardiac remodeling by promoting the polarization of macrophages due to a switch in inflammatory (M1) and reparative (M2) cytokines, i.e., due to the activation of the EP4 (prostaglandin E2 receptor 4) receptor. The reduction in the expression of this enzyme suggests an increase in the number of macrophages with a reparative phenotype and a shift towards reparative signaling and improved post-MI survival (Figure 1) [93].

#### 5.1.10. The Role of miR-22

miR-22 confers a cardioprotective effect against inflammation after a myocardial ischemic/reperfusion injury [94]. In this context, miR-22 overexpression significantly reduces creatine kinase and lactate dehydrogenase serum levels, which are two enzymes that allow for the monitoring of myocardium damage. miR-22 upregulation in the myocardium also decreases infarct size and significantly reduces p38 MAPK, CBP (CREB binding protein), c-Jun-AP-1, and p-c-Jun-AP-1 expression levels. In addition, miR-22 decreases the production of proinflammation mediators such as TNF-α and IL-6 (Figure 1) [94].

#### 5.1.11. The Role of miR-30

miR-30a [95] and miR-30e-5p expression is decreased in in vitro and in vivo myocardial injury [96,97,98]. PTEN (Phosphatase and tensin homolog), a tumor suppressor gene that promotes IL-1, IL-6, and TNF-α production, is a direct target of miR-30e-5p [98,99,100]. On the other hand, miR-30c-5p increased expression in in vitro and in vivo experiments and upregulated Nrf2 (nuclear factor erythroid-2-related factor 2) through the inhibition of the transcription factor Batch1, which is a direct target of miR-30, thus decreasing the production of pro-inflammatory cytokines. Thus, miR-30c downregulates pro-inflammatory cytokine production via Batch/Nrf2 (Figure 1) [96,97]. However, despite this anti-inflammatory role, some authors propose that miR-30c performs a pro-inflammatory function by activating NF-kB signaling via SIRT1. Therefore, additional studies are required to elucidate the dual function of miR-30c in the cardiac inflammatory process.

#### 5.1.12. The Role of miR-499

miR-499 has been associated with a cardioprotective effect due to cytokine production [101] since high levels of this miRNA reduced the expression of TLR2 (Tol-II receptor) in an in vivo model of MI and thus decreased the level of pro-inflammatory cytokines (Figure 1) [102,103,104]. However, the increase in miR-499 in MI upregulates α7-nAchR (alpha-7 nicotinic receptor) expression, increasing the inflammatory response in endothelial cells [105].

#### 5.1.13. The Role of miR-486

Finally, miR-486 was downregulated in an ischemia–reperfusion rat model while in vitro overexpression increased cell viability and reduced inflammation and apoptosis, partly by regulating Foxd3 (forkhead box D3) (Figure 1) [106].

In summary, the NF-kB signaling pathway is primarily modulated by different miRNAs, which play both pro-inflammatory (miR-155, miR-133, miR-130, and miR-23) or anti-inflammatory (miR-181, miR-21, miR-146, miR-24, miR-223, miR-22, and miR-486) roles. Most of these miRNAs are related to the inflammatory process through macrophage polarization and direct cytokine production. Although most of these miRNAs’ roles in cardiac inflammation have been well studied, there are some controversial findings, i.e., those related to miR-30; thus, additional studies are required. Overall, these data highlight the relevant functional role of a large number of miRNAs modulating distinct signaling pathways that ultimately lead to the regulation of the pro-inflammatory and anti-inflammatory responses during cardiac injury. Importantly, the activity of these miRNAs can also be modulated by other non-coding RNAs, such as lncRNAs and circRNAs, thus establishing complex regulatory networks (as summarized in Figure 1).

### 5.2. The Functional Role of miRNAs in Cardiac Fibrosis Response

**Figure 2:**
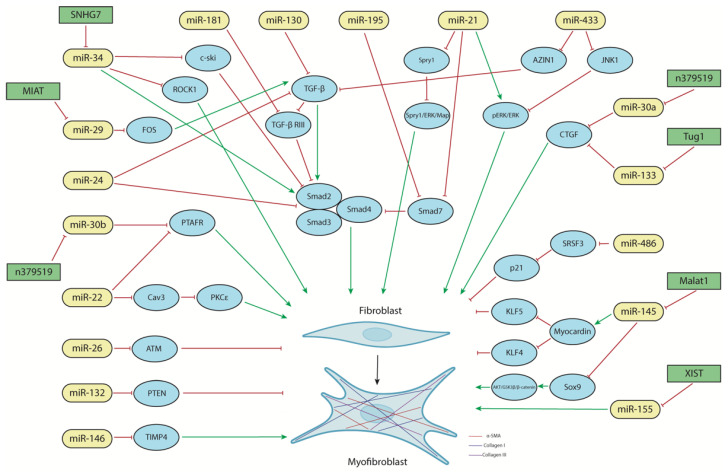
Cardiac fibrosis. Schematic representation of the main miRNAs’ and lncRNAs’ gene-regulatory networks reported to be involved in distinct signaling pathways leading to cardiac fibrosis. Green arrows delineate the ncRNAs that activate the signaling pathways while those depicted in red inhibit such signaling pathways. Note that ncRNAs can influence multiple and distinct signaling pathways leading to the activation of fibroblasts in myofibroblasts and thus ECM deposition. circRNAs are depicted in green, microRNAs are depicted in yellow, and the different signaling pathways modulating the inflammatory response are depicted in blue.

An increasing amount of evidence is also emerging on the functional roles of distinct miRNAs in cardiac fibrosis. Particularly, a handful of miRNAs have been extensively reported in this context, i.e., miR-34, miR-145, miR-181, miR-155, miR-133, miR-22, miR-21, miR-26, miR-29, and miR-30, as described in detail below (Figure 2). Importantly, several of them also have an impact on the inflammatory process, such as miR-181, miR-155, mi2-133, miR-22, miR-21, and miR-30, supporting the notion of the pivotal dual roles of these miRNAs in MI and regeneration (Figure 1 and Figure 2).

#### 5.2.1. The Role of miR-34

**Figure 3:**
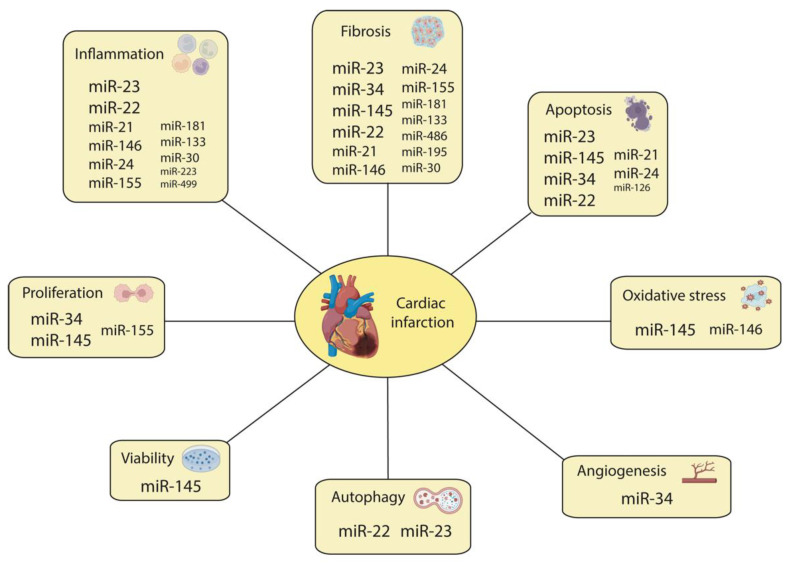
Schematic representation of the distinct miRNAs reported to be involved in distinct biological processes during MI. microRNAs are depicted in large, intermediate, and small sizes according to the number of biological events in which they are involved, e.g., miR-23 is larger since it is involved in inflammation, fibrosis, apoptosis, and autophagy, while miR-30 is only involved in fibrosis. Note that there is increasing information available about the role of microRNAs in three critical biological processes involved in MI, i.e., inflammation, fibrosis, and apoptosis, while the information on their roles in other biological processes, such as proliferation, cell viability, autophagy, angiogenesis, and oxidative stress, is still incipient.

To date, the contributions of miR-34 have exclusively been described in cardiac fibrosis (Figure 1 and Figure 3). Huang et al. [107] reported in vitro and in vivo that miR-34a inhibition reduces fibrosis in the post-MI heart while miR-34 overexpression produces the opposite effects. After Mi was induced in mice, miR-34a was predominantly expressed in the infarcted regions, and collagen I, α-SMA, TGF-β1, and Smad4 were also upregulated. Furthermore, it was determined that TGF-β1 modulated miR-34a during cardiac fibroblasts’ activation since Smad4 is a direct target of miR-34a. Thus, miR-34a can activate the TGF-β1 signaling pathway to induce cardiac fibrosis [107].

Additional evidence on the role of miR-34 in TGF-β1 signaling was more recently reported by Zhang et al. [108] using rat CFs and a rat MI model. In these contexts, miR-34a and miR-93 modulate TGF-β1, inducing fibroblast proliferation and ECM deposition by targeting c-ski and altering CM proliferation (Figure 2) [108]. However, in recent years, Wang et al. [109] have described an anti-fibrotic effect of miR-34-5p, whose expression level was decreased, mediated by the regulation of the lncRNA SNHG7, in the infarcted or peri-infarcted area of cardiac tissues and CFs treated with TGF-β [109]. After MI-induced cardiac fibrosis in mice, lncRNA SNHG7 levels were elevated, CFs’ viability was increased, and the fibroblast–myofibroblast transition was promoted. However, SNHG7 silencing experiments increased miR-34-5p expression (Figure 2), thereby diminishing the promotive effect of ROCK1 (Rho associated protein kinase 1) toward the cardiac fibroblast–myofibroblast transition (Figure 2). Thus, SNHG7 enhances cardiac fibrosis after MI in mice by targeting miR-34-5p and promoting ROCK1 expression, thus inducing CFs′ proliferation and the fibroblast–myofibroblast transition [109].

#### 5.2.2. The Role of miR-145

miR-145 is another microRNA whose action has been defined in the context of cardiac fibrosis following an MI. Although in vitro studies show that hypoxic conditions lead to the downregulation of miR-145 expression in CFs, after miR-145 transient transfection, the number of α-SMA-positive cells is increased, indicating that the conversion of fibroblasts into myofibroblasts has occurred [110,111]. This process is modulated through the KLF5 (Krüppel-like factor 5)-myocardin pathway as miR-145 enhances myocardin expression by inhibiting KLF5, a negative regulator of myocardin (Figure 2) [110,111].

Similarly, in vivo assays reported that three days after MI, miR-145 expression decreases as a result of large-scale fibroblast death and the stimulation of an inflammatory response; however, between days 7 and 14 after MI, miR-145 expression gradually increases, promoting the conversion of fibroblasts into functional contractile myofibroblasts by downregulating KLF4 (Krüppel-like factor 4) until 28 days post-MI (Figure 2) [110]. Furthermore, the in vivo delivery of miR-145 antagomiR one day before MI and two and six days after MI decreases myofibroblast formation and increases scar size (110). Such expression changes suggested that miR-145 might participate in post-infarction remodeling, as it is a necessary factor in the mediation of fibroblast-to-myofibroblast transdifferentiation and the promotion of the migration and polarization of CFs for subsequent scar contraction [111].

In addition to its pro-fibrotic potential, Cui et al. [112] demonstrated that miR-145 can attenuate cardiac fibrosis through the AKT/GSK-3β/β-catenin signaling pathway by directly targeting SOX9 in fibroblasts. The negative regulation of SOX9 mediated by miR-145 in the infarcted rat heart increases PTEN and exerts anti-fibrotic effects via the negative regulation of SOX9, which increases PTEN, thereby inhibiting the PI3K/AKT pathway (Figure 2) [112]. This microRNA also inhibits hypoxic CF survival in neonatal rat CFs and promotes apoptosis via SOX9 and β-catenin downregulation, which was accompanied by a reduction in GSK-3β phosphorylation, proliferation, migration, and collagen synthesis in CFs [112].

The contribution of miR-145 to cardiac fibrosis is also regulated through lncRNAs. Huang et al. [113] reported that MALAT1 lncRNA promotes cardiac fibrosis and deteriorates cardiac function post-MI in mice by regulating TGF-β1 activity via miR-145 (Figure 2) [113]. At the cellular level, MALAT1 knockdown promotes AngII-induced neonatal mouse CF proliferation, collagen production, and myofibroblast transdifferentiation [113]. In both approaches, MALAT1 lncRNA directly regulates miR-145, acting as a sponge of this microRNA that can enhance TGF-β1 activity and increase the expression of Furin, of which the latter promotes cardiac fibrosis [113]. Overall, these data support the idea that miR-145 has a dual role in cardiac fibrosis.

#### 5.2.3. The Role of miR-181

Indirect evidence on the role of miR-181 in cardiac fibrosis was reported by Vaskova et al. [114] who observed that the downregulation of miR-181a results in the attenuation of myocardial fibrosis and hypertrophy, thereby restoring an injured rodent heart after MI [114]. Additional evidence was reported by Chen et al. [115] who demonstrated that miR-181 upregulation in an MI rat model is associated with ECM deposition, a process that can be downregulated using microRNA inhibitors, partly by targeting TGF-β receptor III (TβRIII) (Figure 2) [115]. However, the molecular mechanisms that contribute to the attenuation of myocardial fibrosis have not yet been fully elucidated.

#### 5.2.4. The Role of miR-155

After myocardial injury, miR-155 not only regulates the inflammatory process but also participates in the proliferation and differentiation of myofibroblasts after myocardial injury, performing a pro-fibrotic role [42,116]. In a recent study using mice with dyslipidemia and without the expression of miR-155 (Apo E^−/−^, miR155^−/−^), the fibroblast density in the post-ischemic scar was increased after MI was significantly reduced [117]. Such an effect of miR-155 was corroborated by an inhibition of miR-155-downregulated α-SMA in fibroblasts and decreased myofibroblast differentiation and proliferation (Figure 2) [118,119,120]. Nevertheless, an opposite role of this microRNA has been described in an in vitro assay on human CFs where the interaction between lncRNA XIST and miR-155 downregulates microRNA expression, producing high levels of fibrotic proteins such as Col1α1, Col1α3, and α-SMA and regulating the proliferation and apoptosis of fibroblasts (Figure 2) [121]. Therefore, miR-155 seems to play a dual role in fibrosis. Additional studies will be required to elucidate the triggering causes of its pro-fibrotic vs. anti-fibrotic effects.

#### 5.2.5. The Role of miR-133

The role of miR-133 during cardiac fibrosis has been reported in several distinct experimental approaches. Dakhlallah et al. [122] demonstrated that miR-133a-transfected mesenchymal stem cells administered to a mouse model of MI led to increased cell engraftment, decreased fibrosis, improved cardiac function, and reduced expression of proapoptotic genes [122]. Yu et al. [123] demonstrated a reduction in miR-133a levels in the infarct border zone after MI in mice and that forced miR-133a expression improves cardiac function and reduces fibrosis, providing evidence that the expression of TGF-β1, CTGF (connective tissue growth factor), Col1α1, Col1α3, and α-SMA were decreased (Figure 2) [123]. Additional evidence on the role of miR-133 was reported in a study by Zhu et al. [124] wherein miR-133a-3p-containing exosomes enhanced proliferation, migration, and angiogenesis while inhibiting CM apoptosis in vitro in H9c2 CMs and HUVEC cells, respectively [124]. Furthermore, miR-133a-3p-enriched exosomes also significantly inhibit CM apoptosis, reduce fibrotic area, and improve cardiac function as measured via echocardiography in infarcted rats in vivo by modulating Akt phosphorylation [124]. The role of miR-133 in cardiac fibrosis, particularly miR-133b, is also modulated by lncRNAs; for instance, complementary patterns of TUG1 (taurine-upregulated gene 1) lncRNA, and miR-133b were identified in a rat model of myocardial fibrosis. TUG1 lncRNA knockdown decreases myofibroblast activation, while forced expression increases proliferation and collagen deposition by acting through miR-133 and its downstream targets, i.e., CTGF (Figure 2) [118]. In sum, these studies demonstrate that miR-133 exerts anti-fibrotic effects during MI.

#### 5.2.6. The Role of miR-22

The study of the functional role of miR-22 in cardiac fibrosis has generated controversial results. Hong et al. [125] described miR-22 as a negative regulator of cardiac fibrosis following induced MI in mice and angiotensin II treatment in CFs due to its effects on collagen deposition and Col1α1, Col1α3, and TGFβR1 expression (Figure 2) [125]. However, a pro-fibrotic role of miR-22 was identified in similar experimental models in which this microRNA accelerated cardiac fibrosis through the miR-22–Cav3–PKCε pathway, inducing the proliferation and differentiation of CFs into myofibroblasts and collagen deposition due to the activation of PKCε (Figure 2) [126]. More recently, in CFs isolated from mouse hearts treated with Angiotensin II, miR-30-5p and miR-22-3p were downregulated, whereas the levels of PTAFR (platelet activating factor receptor) were increased [127]. miR-22-3p mimic transfection reduces the expression of proliferation markers, such as cyclin D1 and PCNA (proliferating cell nuclear antigen), as well as collagen-deposition-inhibiting Col1α1, Col1α3, and α-SMA expression [127]. It also represses the mRNA and protein levels of PTAFR, which decrease CFs viability, suggesting an anti-fibrotic effect mediated by miR-22-3p in AngII-treated CFs (Figure 2) [127]. Therefore, additional efforts are required to fully elucidate the plausible dual role of miR-22 in cardiac fibrosis.

#### 5.2.7. The Role of miR-21

Despite the anti-inflammatory effect of miR-21 after MI, this miRNA has also been implicated in fibrosis, in which it plays a pro-fibrotic role [128,129]. In plasma samples taken from MI patients, the levels of miR-21 increased over time after cardiac injury, and the same result was observed in the ischemic area after coronary artery ligation in mice [128]. Furthermore, in vitro TGF-β-treated CFs promoted increased expression of miR-21, col1α1, α-SMA, and F-actin [128]. A luciferase assay demonstrated that Smad7, a negative regulator of TGF-β factor, is a target of miR-21, thus providing further evidence of a role of miR-21 in fibrosis via the regulation of the TGF-β/Smad7 pathway (Figure 2) [128,130]. miR-21 also regulates other signaling pathways, such as pERK/ERK and Spry1/Erk/Map, with the latter contributing to fibrosis (Figure 2) [131,132]. In H9c2 cells, the expression of TGF-β and miR-21 was increased, establishing that miR-21 promotes fibrosis by regulating TGF-β/Smad3 signaling [133]. Therefore, miR-21 has anti-inflammatory and pro-fibrotic roles because it participates in inflammation by inhibiting the p38 and NF-kB pathways, thus decreasing pro-inflammatory cytokine production, and in fibrosis by downregulating TGF-β pathway inhibitors (Figure 2).

#### 5.2.8. The Role of miR-26

In both cell cultures, namely, oxygen-glucose-deprivation (OGD)-treated CMs and H9c2 cells and an in vivo MI mouse model, miR-26a overexpression decreases collagen 1, CTGF, and ATM (Ataxia-telangiectasia mutated) expression levels, reducing cardiac fibrosis and apoptosis [134]. miR-26b is another member of the miR-26 family that plays a role in MI. This miRNA negatively targets PTGS2 (prostaglandin-endoperoxidase synthase 2), activating the MAPK signaling pathway and thus reducing the inflammatory response and improving myocardial remodeling after MI in mice (Figure 2) [135].

#### 5.2.9. The Role of miR-29

Several studies suggest that miR-29 family members can target mRNAs involved in fibrosis, such as collagens, fibrillins, and elastin [136]. In vitro assays in CFs have reported that miR-29a overexpression decreases the expression of FOS, thereby reducing cell proliferation and migration induced by TGF-β1 [137]. The implication of the miR-29 family in fibrosis has also been studied in animal models with hypertension; for instance, the overexpression of miR-29b in a mouse heart prevents AngII-mediated cardiac fibrosis and cardiac dysfunction by targeting the TFG-β/Smad3 pathway (Figure 2) [138]. In another pathological context, as with MI, miR-29b overexpression in rat myocardial tissues significantly improves cardiac function by reducing collagen volume fractions and downregulating the expression of Col1α1 and α-SMA through the targeting of SH2B3 (SH2B adapter protein 3) [139]. Curiously, the ability to attenuate fibrosis after an MI via targeting the TGF-β/Smad3 pathway with several drugs, such as carvedilol, tanshinone IIA, and leonurine, is associated with miR-29 overexpression. The cardioprotective effect of these drugs is lost if an miR-29 antagonist is administered [140,141,142]. Furthermore, this role as a fibrogenic repressor is shared by another member of the miR-29 family, miR-29a, which is regulated by lncRNA MIAT in human patients with hypertrophic cardiopathy (Figure 2) [143].

#### 5.2.10. The Role of miR-30

Several members of the miR-30 family are involved in cardiac fibrosis after MI, such as miR-30a, miR-30b-5p, and miR-30d. It has been demonstrated that miR-30a directly regulates CTGF, inhibiting its expression and reducing cardiac fibrosis (Figure 2) [144,145]. The fibrotic process and collagen production are also related to miR-30b-5p, which has been reported to be downregulated in cardiac fibroblasts from rat hearts that were treated with angiotensin II to induce MI; at the same time, an upregulation of PTARF (platelet-activating factor receptor) was also reported [127]. This situation produces an increase in fibroblast proliferation and collagen deposition, suggesting that miR30b-5p controls fibrogenesis by regulating PTAFR (Figure 2) [127]. In rats with HF, CMs’ production of EVs enriched with miR-30d downregulates fibroblast proliferation and reduces fibrosis. However, reduced levels of miR-30d in a chronic phase are related to increased fibrosis and inflammation markers, suggesting that the effect of the miR-30 family in fibrosis depends on the phase of the cardiac injury [146]. The MiR-30 family can also be regulated by other non-coding RNAs, for instance, lncRNAs. In an MI study using murine models, high levels of n379519 lncRNA, which negatively regulates miR-30, were associated with lower levels of fibrosis and collagen synthesis (Figure 2) [147].

Besides those miRNAs that have been widely documented to play pivotal roles in cardiac fibrosis, there is emerging evidence for the involvement of an additional handful, e.g., miR-24, miR-433, miR-146, miR-486, miR-132, miR-130, and miR-195; some of which also modulate the inflammatory response (i.e., miR-24, miR-146, miR-130, and miR-486) during cardiac injury (Figure 1, Figure 2 and Figure 3).

#### 5.2.11. The Role of miR-24

Several in vitro and in vivo studies have reported that an upregulation of miR-24 reduces the proliferation and differentiation of CFs through the downregulation of TGF-β, which, in turn, occurs through the action of Furin and Smad2/3 phosphorylation (Figure 2) [148].

#### 5.2.12. The Role of miR-433

miR-433 was consistently elevated in three models of heart disease with prominent cardiac fibrosis, and it was enriched in fibroblasts compared to CMs. miR-433 forced expression in neonatal rat CFs enhances proliferation and differentiation into myofibroblasts through the targeting of AZIN1 (antizyme inhibitor 1) and JNK1. AZIN1 downregulation promotes TGF-β1 activation, while JNK1 downregulation leads to ERK and p38 kinase activation, thus promoting cardiac fibrosis (Figure 2) [149].

#### 5.2.13. The Role of miR-146

Several studies, both in vitro and in vivo, have reported that miR-146 promotes fibroblast proliferation and myofibroblast differentiation. Furthermore, MI mice treated with anti-miR-146b displayed high levels of TIMP4 (TIMP metallopeptidase inhibitor 4) and showed a significant reduction in fibrosis in the injured zone (Figure 2) [150]. In the same way that miR-146 and miR-155 have a synergic role in the context of inflammation, a combined mode of action for miR-146 and miR-126 has also been described in fibrosis. MI rats treated with exosomes derived from adipose mesenchymal stem cells containing miR-146 and miR-126 showed less fibrosis and more collagen fibers [151].

#### 5.2.14. The Role of miR-486

Chen et al. [152] demonstrated that miR-486 targets SRSF3 (serine/arginine-rich splicing factor 3)/p21 and thereby mediates cardiac myofibroblasts’ senescence (Figure 2). Thus, an improvement in their fibrotic activity was observed, which benefits the regeneration of MI by limiting scar size and post-MI remodeling in mice [152].

#### 5.2.15. The Role of miR-132

In cardiac progenitor cells cultured under hypoxia, exosomes with miR-132 have been reported to reduce the expression of fibrotic proteins and stimulate angiogenesis [153]. Furthermore, miR-132 overexpression in a rat MI model and in AngII-treated CFs decreases the expression levels of col1α1, col1α3, TGF-β, and α-SMA. miR-132 inhibits PTEN expression, a key gene involved in PI3K/Akt signal pathway, thereby decreasing cardiac fibrosis (Figure 2) [154].

#### 5.2.16. The Role of miR-130

In mouse CFs cultured under hypoxic conditions and after MI, miR-130a expression decreases, whereas the expression of TGF-β, α-SMA, col1α1, and TGF-β receptor 1 (TGFBR1) increases. In contrast, overexpression of miR-130a decreases the expression of these factors and reduces the area of cardiac fibrosis, thus improving cardiac function in post-MI mice [155].

#### 5.2.17. The Role of miR-195

Secreted by injured CMs within cardiosomes in a murine model of MI, miR-195 can activate primary isolated CFs triggering, via the targeting of SMAD7, the transcription of collagen, FAP (fibroblast activation protein), fibronectin ED-A, CXCL1, and IL-6, promoting the activation of α-SMA and leading to myofibroblast differentiation (Figure 2) [156]. Thus, miR-195-enriched cardiosomes activate cardiac myofibroblasts [156].

As mentioned above, several miRNAs participate in cardiac fibrosis regulation, which is a complex event characterized by increased fibroblast proliferation and ECM deposition. Some of them have been shown to favor fibrogenesis, among which are profibrotic miRNAs such as miR-21, miR-181 miR-195, miR-24, miR-433, or miR-146, whereas others, including miR-133, miR-26, miR-29, miR-30, miR-486, miR-132, or miR-130a, have been reported to have an anti-fibrotic role. In addition, it is important to consider that the posttranscriptional regulations mediated by miRNA are so complex that some of these miRNAs, e.g., miR-34, miR-145, miR-155, or miR-22, seem to have a dual role in cardiac fibrosis.

### 5.3. The Role of miRNAs in Other Biological Processes during MI

While it is highly documented that inflammation and fibrosis represent the paramount hallmarks of cardiac infarction, additional concomitant and intermingled biological processes such as apoptosis, cell proliferation, ROS modulation, and angiogenesis, which number among the most representative events, also occur during cardiac infarction. A large body of evidence has demonstrated that miRNAs are also involved in these processes, namely, miR-145, miR-34, miR-23, miR-126, miR-146, miR-22, miR-21, miR-155, miR-24, miR-208, and miR-590. Among them, apoptosis has been reported to be modulated by a large number of miRNAs (miR-145, miR-23, miR-34, miR-22, miR-126, miR-21, and miR-24), as detailed below (Figure 3). Cell proliferation is also modulated by distinct miRNAs such as miR-145, miR-34, and miR-126 associated with different cardiovascular cell types, while ROS signaling and angiogenesis are modulated by miR-145, miR-146, and miR-34, respectively (Figure 3).

Importantly, many of these miRNAs are also implicated during cardiac inflammation (miR-23), cardiac fibrosis (miR-145), or simultaneously in both events (miR-155, miR-21, miR-146, miR-24, and miR-22), supporting the notion of a pivotal role of these miRNAs with respect to orchestrating different aspects of cardiac infarction (Figure 3). In the next few paragraphs, we will summarize the current information on the functional roles of these miRNAs, ranging from those modulating multiple processes during cardiac infarction to those that, to date, have only been involved in discrete processes.

#### 5.3.1. The Role of miR-145

Several studies have identified a cardioprotective role of miR-145-5p through both in vitro and in vivo ischemic approaches. For instance, in a model of myocardial hypoxic/ischemic injury, after exposing H9c2 cardiac cells to hypoxia, miR-145-5p was notably downregulated, while CD40, inflammatory response cytokines, and apoptotic genes were highly expressed. However, miR-145-5p mimic transfection effectively suppressed CD40 expression, decreased inflammatory factor production, and significantly augmented the expression of Bcl-2 [157]. Similarly, in vitro assays reported a significant increase in miR-145 expression, which was dependent on HIF-1α overexpression. miR-145 overexpression was positively related to cell viability, that is, the protection of CMs against apoptosis, i.e., the inhibition of the expression levels of cleaved caspase-9, cleaved caspase-3, and cleaved PARP while increasing Bcl-2 expression, via the modulation of SGK1 (serum/glucocorticoid regulated kinase 1) and some factors of the PI3k/AKT signaling pathway. However, in a rat MI model, the significantly increased expression levels of miR-145 and SGK1 suggest that SGK1 signaling might occur in parallel with the miR-145 effect [158]. After rat heart infarction, miR-145 overexpression promotes left ventricular systolic function improvement and significantly reduces the size of infarcted myocardial tissues [159]. Furthermore, miR-145 overexpression decreases the levels of the pro-apoptotic protein Bax and that of cleaved caspase-3 and increases the expression of the anti-apoptotic protein Bcl-2, thereby reducing the level of CM apoptosis induced by MI. Importantly, PDCD4 (programmed cell death protein 4), an upregulated gene during apoptosis, is a direct target of miR-145 in CMs. In hypoxia-treated CMs, miR-145 overexpression causes a reduction in apoptosis through the attenuation of mitochondrial dysfunction after targeting PCDC4. Thus, the overexpression of miR-145 has a cardioprotective function against rat MI through the targeting of PDCD4 [159].

In vitro and in vivo assays demonstrated that miR-145-5p mimics could induce a reduction in NADPH oxidase homolog 1 (NOH-1) levels and decrease the levels of TNF-α, IL-1β, and IL-6 by stimulating oxygen and glucose deprivation/reperfusion (OGD/R), increasing SOD activity, and reducing ROS [160]. Likewise, miR-145 mimic transfection of murine embryonic CMs in a model of hypoxia/reperfusion significantly increased cell proliferation, improved the release of SOD, upregulated ERK1/2 and p-AKT, and downregulated IL-1β, IL-6, P38MAPK, p53, and Bax. Furthermore, the overexpression of miR-145-5p downregulated GIGYF1 (GRB10 interacting GYF protein 1), a direct target of this microRNA, thereby promoting cell proliferation, inhibiting cell apoptosis, and alleviating inflammation and oxidative stress. These results were also confirmed in an ischemia–reperfusion rat model, suggesting that miR-145-5p plays a protective role in MI [161].

All these data highlight the role of miR-145 in apoptosis, cell proliferation, and cell viability; its involvement in ROS modulation and mitophagy; and its previously mentioned role in inflammation and fibrosis, suggesting that this miRNA is pivotal for multiple biological processes during cardiac infarction.

#### 5.3.2. The Role of miR-34

After birth, miR-34a expression is associated with a loss of regenerative potential, which reduces the protein levels of Sirtl, Cyclin D1, and Bcl2. Additionally, in adult rat MI models, the overexpression of miR-34a promotes CM cell death, reduces CM proliferation, and enhances tissue fibrosis [162]. Furthermore, miR34a expression is increased in aging mouse hearts following acute MI, aggravating hypertrophy, fibrosis, CM senescence, and apoptosis. In vitro and in vivo assays have shown that the inhibition of miR-34a reverts those effects and inhibits age-related and MI-induced CM cell death. The inhibition of miR-34a in the heart upregulates PNUTS (protein phosphatase-1 nuclear targeting subunit), which reduces telomere erosion, cardiac DNA damage response signaling, and CM apoptosis after acute MI. Therefore, during aging and after acute MI, miR-34a regulates cardiac contractile function by directly targeting PNUTS [163].

Another role for miR-34 is the modulation of endothelial tube formation. High-glucose-insulted, human-bone-marrow-derived mesenchymal stem cells transfected with miR-34c and injected into the peri-infarcted area after MI induction diminished the angiogenic activity of BMCs due to their disability to form vasculature. miR-34c silences SCF (Stem cell factor) and promotes KLF4 induction, which altered tube formation [164]. Thus, miR-34 can functionally modulate both CMs and endothelial cell behavior in the context of MI.

#### 5.3.3. The Role of miR-23

In myocardial ischemia–reperfusion-injured rats and hypoxia/reoxygenation-exposed 293T cells, miR-23a expression is upregulated while the levels of CX43 (connexin 43), a direct target of this microRNA, are remarkably decreased, correlating with autophagy activation and significantly reduced cell viability. Importantly, miR-23a overexpression leads to the upregulation of protein markers of mitophagy, such as PINK1 (PTEN-induced kinase 1), Parkin, or LC-3. Thus, miR-23a, by directly targeting CX43, enhances mitophagy in cases of myocardial ischemia/reperfusion injury [165]. In the context of cardiac infarction, however, indirect roles of miR-23a and miR-92a have been reported. BM-MSC transplantation in rat cardiac infarction models significantly reduces fibrosis, apoptosis, and miR-23a and miR-92a expression. BM-MSC media, after exposure to hypoxic conditions, display higher levels of VEGF (Vascular endothelia growth factor), MCP-1 (Monocyte chemoattractant protein 1), IL-6, and ANG (Angiogenin). When hypoxia-exposed neonatal rat CMs were treated with hypoxia-exposed BM-MSC-conditioned media, apoptosis was reduced, and miR-23a and miR-92a were downregulated. Furthermore, the neutralization of VEGF, a paracrine factor present in hypoxia-exposed BM-MSC-conditioned media, increases the rates of apoptosis in CMs and miR-23a and miR-92a expression. Therefore, VEGF, derived from transplanted bone-marrow-derived mesenchymal stem cells, plays an anti-apoptotic role by regulating miR-23a and miR-92a in CMs after MI [166].

#### 5.3.4. The Role of miR-126

The protective role of exosomes loaded with miR-126 from adipose-derived stem cells (ADSC) has been analyzed in vitro and in vivo. In both cases, miR-126-enriched exosomes reduced the expression of inflammatory cytokines, decreased the expression of fibrosis-related protein, and promoted angiogenesis. In addition, these exosomes prevented CM apoptosis and promoted cell proliferation. Thus, miR-126-enriched, ADSC-derived exosomes protect myocardial cells from apoptosis, inflammation, and fibrosis while promoting angiogenesis [167].

#### 5.3.5. The Role of miR-146

The role of miR-146a has also been studied in mice and rat CMs subjected to ischemia–reperfusion [168]. In mice, fibrosis and the size of the injured area were simultaneously reduced alongside inflammation and ROS after miR-146a overexpression, while in rat CMs administered an anti-miR146a treatment, the opposite effects were observed. miR146a directly regulates NOX4 (NADPH oxidase 4), a NOX family enzyme that produces ROS [169], leading to the inhibition of p38 phosphorylation in the MAPK pathway [168], which is related to the anti-inflammatory effect [170].

#### 5.3.6. The Role of miR-22

In an ischemia–reperfusion injury experimental rat model, miR-22 directly targeted CREB binding protein (CBP), which attenuates p53 acetylation, leading to the downregulation of p21 and Bax. Therefore, miR-22 could inhibit CM apoptosis by targeting CBP [171].

#### 5.3.7. The Role of miR-21

As is the case with other miRNAs, miR-21 is also transported in exosomes [172,173]. In cardiac telocytes, exosomes with miR-21 were identified, which were directly regulating Cdip1 (cell death inducing p53 target 1) and inhibiting p53 expression. Such effects cause decreased apoptosis in endothelial cells after MI [174]. Another study confirmed the protective effect of the serum after MI mediated by extracellular vesicles carrying miR-21 since miR-21 downregulation leads to increased apoptosis in CMs through the regulation of PDCD4 expression [175,176]. miR-21 is also regulated by lncRNAs such as GAS-5 lncRNA, which regulates apoptosis in CMs after MI by sponging miR-21. In H9c2 cells, the inhibition of GAS-5 induces the downregulation of PI3K/AKT and an increase in the rate of apoptosis. Furthermore, in an MI rat model, GAS-5 lncRNA and PDCD4 levels were downregulated while miR-21 was over-expressed, thereby promoting greater fibrosis and collagen synthesis. Therefore, through miR-21, GAS-5 controls PCDC4 expression, which regulates the apoptosis of CMs [177]. Additional evidence of an miR-21/GAS-5 interaction was also reported in rats with MI induced by an isoproterenol treatment [178]. Thus, miR-21 seems to decrease levels of apoptosis in endothelial cells after MI, while miR-21 overexpression mediated by GAS5 downregulation increases the levels of CM apoptosis and fibrosis.

#### 5.3.8. The Role of miR-155

In mice, miR-155 gain- and loss-of-function assays revealed a negative relationship between this miRNA and TP53INP1 (tumor protein p53-inducible nuclear protein 1). In addition, TP53INP1 promoted fibroblasts’ proliferation and conversion into myofibroblasts, thereby supporting the notion that miR-155 enhances fibrosis [56]. However, such mechanisms have not been described in the context of MI.

#### 5.3.9. The Role of miR-24

In CMs and fibroblasts, low levels of miR-24 have been reported, which were associated with increased apoptosis. On the other hand, the levels of miR-24 increase in endothelial cells after MI [179]. Furthermore, in another report, CM apoptosis was reduced by using exosomes with high levels of miR-24 cargo, thereby improving cardiac function [180]. However, the molecular mechanism by which miR-24 influences apoptosis in both cell types remains to be elucidated.

#### 5.3.10. The Role of miR-208

miR-208a expression levels are increased in conditions of oxidative stress, fibrosis, and inflammation after MI. An in vitro assay using H9c2 cells cultured under hypoxia showed that miR-208 and TGF-β levels increase with time during incubation, which is in line with similar findings in MI patients [133,181]. Additionally, in a healthy heart, miR-208a overexpression promotes the expression of endoglin, a membrane glycoprotein of the TGF-β receptor signaling pathway, whereas in an MI rat model, particularly high levels of this miRNA can be observed in the fibrotic area [182].

#### 5.3.11. The Role of miR-590

miR-590 administration after MI in neonatal and young mice reduces the infarction area, recovers cardiac function, and decreases fibroblast proliferation, while CM proliferation is increased. Mechanistically, miR-590 targets the HOP homeobox, a transcription factor that inhibits CM proliferation and chloride intracellular channel 5 (CLIC5), of which the latter is an inhibitor of cell proliferation [183,184].

### 5.4. The Role of miRNAs during Cardiac Regeneration

After a cardiac injury, the heart is only capable of naturally healing in a subset of species, as previously stated. Throughout this process, inflammation and fibrosis play crucial roles in clearing out and stabilizing cardiac morphology and function. However, healing requires additional processes, such as cell proliferation and scar resolution. The comparison of species with and without cardiac regeneration capacity provides an ideal basis from which to search for molecular mechanisms that can serve as therapeutic tools to heal dysfunctional hearts without an innate regenerative capacity.

Several experimental models have provided insights into the cellular and molecular mechanisms that govern cardiac regeneration. In addition, as detailed in the following paragraphs, within this context, several miRNAs have been identified to play essential roles in cardiac regeneration with respect to processes such as inflammation, fibrosis, angiogenesis, and apoptosis.

#### 5.4.1. The Role of miR-195

One of the most representative microRNAs studied with regard to cardiac regeneration is miR-195. In one study, mice overexpressing miR-195 developed congenital heart defects, including ventricular septal defects, ventricular hypoplasia, and a reduction in ventricle weight [185]. At P1, transgenic hearts overexpressing miR-195 showed a reduction in the number of cells undergoing mitosis and an increased proportion of multinucleated myocytes. These data indicate that the in vivo overexpression of miR-195 inhibits CM mitotic progression and induces premature cell cycle arrest by suppressing the expression of cell cycle genes such as checkpoint kinase 1 (Chek1). In addition, the overexpression of miR-195 prevents heart regeneration post-MI in P1 mouse hearts when cardiac tissue regeneration is still possible. In cell cultures, using both myoblastic cell line H9c2 and neonatal rat CMs, miR-195 overexpression was found to be associated with cellular hypertrophy and a greater proportion of binucleate cells. Furthermore, cells that expressed miR-195 were accumulated in the G2 phase, suggesting that this microRNA blocks the G2/M phase transition, thereby altering mitotic progression. Therefore, miR-195 is upregulated in the postnatal (1–10 days of age) ventricular chambers at the time of the mitotic arrest of CMs in mice. Thus, it is probably involved in the transition to quiescence [184,185].

#### 5.4.2. The Role of miR-126 and miR-146

Evidence on the role of miR-146 in cardiac regeneration has been obtained by exosome-mediated delivery. Using an MI rat model, Shafei et al. [151] demonstrated that miR-126- and miR-146a-loaded exosome injections, derived from mesenchymal adipose stem cells, do not reduce infarct size and fibrosis but promote angiogenesis, upregulating CD31 and CX43 expression in the infarcted area. Furthermore, in vitro cultures of HUVECs demonstrated that exosomes loaded with miR-126 and miR-146a improve cell proliferation, cell migration, and tube formation and induce VEGF expression. Thus, the co-administration of miR-146 and miR-126 in isolated exosomes promoted vascular integrity and reduced infarct size in an MI model [151]. Importantly, additional evidence on the angiogenic capacities of miR-126 has also been reported in other biological systems [186,187].

#### 5.4.3. The Role of miR-98

Additional evidence on the role of microRNAs loaded into exosomes has been reported. The injection of miR-98-5p-containing exosomes in vivo leads to the recovery of oxidative stress, inflammation, and infarct size. Furthermore, Zhang et al. [188] reported that miR-98-5p is downregulated in myocardial ischemia/reperfusion rat myocardial tissue. However, an administration of hypoxia-induced bone marrow mesenchymal stem cell (BMSC)-derived exosomes containing miR-98-5p enhanced cardiac function by suppressing myocardial enzyme levels, oxidative stress, inflammatory responses, macrophage infiltration, and infarct size, modulating PI3K/Akt and TLR4 signaling [188]. Thus, these data demonstrate the role of miR-98-5p in distinct biological processes involved in MI and cardiac regeneration.

#### 5.4.4. The Role of miR-22

Cardiac miR-22 expression levels are increased in aging mice and cultured neonatal CMs through a p53-dependent mechanism. In vitro inhibition of miR-22 in aging CMs and in vivo prevents post-infarction remodeling and improves cardiac function. On the other hand, miR-22 overexpression increases the quantity of p62 aggregates, providing evidence of a role of these microRNAs as regulators of cardiac autophagy [189].

#### 5.4.5. The Roles of miR-1 and miR-29

A microarray transcriptomic analysis of an MI mouse model identified differentially expressed miRNAs that, if overexpressed, modulate myocyte growth, fibrosis, and inflammation. In particular, a gene network analysis identified miR-1, miR-29b, and miR-98 as key agents in MI. miR-1 regulates myocyte growth, while miR-29b and miR-98 are key agents in fibrosis and inflammation, respectively [190].

Furthermore, evidence of the role of circRNA-modulated miRNAs has been reported using in vitro hypoxia/reperfusion models as well as in vivo [191,192]. In an in vitro model of hypoxia/reperfusion, the administration of exosomes with high amounts of circ_0001747 partially recovered CM dysfunction by targeting the miR-199b/MCL1 (induced myeloid leukemia cell differentiation protein) signaling pathway. Exosomes with high levels of circ_0001747 attenuate in vitro hypoxia/reperfusion-induced HL-1 dysfunction partly by targeting miR-199b-3p/MCL1 signaling [191]. Similarly, lncRNA-modulated miRNAs are also important in this context. Within hydrogen-peroxide-induced acute MI cell models and acute MI mice, TTTY15 lncRNA is upregulated while miR-98-5p is downregulated. The knockdown of TTTY15 lncRNA alleviates myocardial cell injury in vitro and AMI progression in vivo, processes modulated by sponging miR-98-5p [192].

In addition, macrophage-derived EVs containing lncRNAs that modulate miR-25-3p function can negatively influence MI recovery. In addition, M1 macrophage-derived EVs containing MALAT1 lncRNA can competitively bind to miR-25-3p and thus inhibited angiogenesis and myocardial regeneration in a mouse model of MI. MALAT1 sponges miR-25-3p and upregulates CDC42 (cell division control protein 42 homolog). Importantly, miR-25-3p overexpression promotes cell viability, proliferation, and angiogenesis [193].

## 6. LncRNAs in Cardiac Infarction and Regeneration

LncRNAs are structurally similar to mRNAs since they are transcribed by RNA polymerase II and have the same typical post-transcriptional modifications, i.e., a 5’ terminal cap and a 3´ terminal poly (A) tail, yet they lack the capacity to encode proteins. Mechanistically, they can act both as transcriptional regulators, modulating nuclear gene expression in different ways such as epigenetic landscape control, transcriptional complex scaffolding, or using decoy molecules, or as post-transcriptional regulators modulating microRNA degradation, mRNA stability, and/or protein translation. It is worth mentioning that the wide variety of functions that lncRNAs can perform may reflect the importance of this class of RNAs in the regulation of multiple biological processes, including cardiac injury and regeneration.

### 6.1. The Role of lncRNAs in Cardiac Inflammation

It has been suggested that many different lncRNAs play essential roles in cardiac inflammation. Several lncRNAs modulate the expression of miRNAs that have been widely reported to affect key signaling pathways involved in the inflammatory response, as previously described in the preceding paragraphs. However, an increasing number of lncRNAs are also being described as capable of modulating the expression of distinct miRNAs with limited involvement in cardiac inflammation, as summarized herein (Table 1).

In this context, several lncRNAs can modulate miRNAs and play essential roles in cardiac inflammation, such as KCNQ1OT1 lncRNA, which sponges miR-130 and thus modulates ZNF91 (zinc finger protein 91), and Lucat1 lncRNA, which sponges miR-181. However, the molecular mechanisms relating them to cardiac inflammation remain largely elusive [40,83]. Additionally, other lncRNAs modulating miRNAs have emerging roles in cardiac inflammation, such as HULC lncRNA, which modulates the expression of miR-29 and miR-377, leading to the regulation of the NLRP3/Caspase-1/IL-1β signaling pathway, while TTTY15 lncRNA modulates miR-98 and, thereafter, CRP expression [192,194,195]. Furthermore, several other lncRNAs have also been implicated in cardiac inflammation due to their modulation of microRNA expression, such as MIAT2/miR-15 and MIRT2/miR-377 [196,197]. However, in these cases, the downstream signaling pathways have yet to be fully characterized. On the other hand, Gm2691 directly affects Akt signaling and, consequently, inflammation, whereas MIRT1 lncRNA influences NF-kB signaling [198,199]. Finally, three additional lncRNAs, namely, NEAT1, LUNAR1, and SNHG8, have been implicated in cardiac inflammation, although the molecular mechanisms underlying their effects remain to be elucidated [200,201,202].

### 6.2. The Role of lncRNAs in Cardiac Fibrosis

Similarly, an increasing number of lncRNAs are reported to modulate cardiac fibrosis in the MI context. Several of these lncRNAs have been used to identify partner proteins via pull-down assays. For example, Safe lncRNA interacts with Sfrp2 (secreted frizzled related protein 2) and HuR (human antigen R), Wisper interacts with Tia1-related protein, Sail lncRNA interacts with Safb (scaffold attachment factor B), and Cfast interacts with Colt1 and Trap1 (TNF-receptor-associated protein 1) [203,204,205,206]. In all these cases, these lncRNAs are critical regulators of signaling pathways that are involved in fibroblast/myofibroblast activation and thus extracellular matrix deposition.

The modulation of distinct signaling pathways by the microRNA sponging of these lncRNAs has also been reported. Pcfl lncRNA modulates miR-378 and thus Grb2 (growth factor receptor bound protein 2) expression, while Norad lncRNA sponges miR-577, resulting in the modulation of Cobl1 (cordon-bleu protein-like 1), in both cases altering collagen synthesis [207,208]. In addition, Plf regulates let-7d and, thereafter, Ptafr [209].

Several other lncRNAs modulate the expression of distinct miRNAs that influence signaling pathways leading to fibrosis, such as Mhrt/miR-3185, n379519/miR-30, and Xist/miR-155, although the precise affected signaling pathways remain unclear [121,147,210]. Finally, a series of lncRNAs are reported to modulate TGF-β1/Smad signaling, such as lncRNA554, Lnc-Ang362, LncR-30245, and NILR, yet the molecular mechanisms remain to be elucidated [211,212,213,214]. Importantly, FGD5-AS1 lncRNA can modulate both processes, i.e., inflammation and fibrosis, through miR-223 sponging and thus the modulation of Akt signaling [91]. 

### 6.3. The Role of lncRNAs in Other Biological Processes of Cardiac Infarction

In line with the reports previously mentioned, several lncRNAs can impact distinct processes such as myocardial apoptosis and ROS signaling; moreover, in some cases, they also affect cardiac inflammation and fibrosis. In this context, Liu et al. [215] described that Mirt1 can directly affect NF-kB signaling, leading to the regulation of fibrosis, inflammation, redox balance, and apoptosis, even though the molecular mechanisms driving such effects remain largely elusive. Moreover, Gas5 modulates miR-21, leading to myocardial apoptosis, while LNC_000898 lncRNA modulates miR-375 and thus Pdk1, leading to cardiac apoptosis [178,216].

### 6.4. The Role of lncRNAs in Cardiac Regeneration

Several lines of evidence have demonstrated the pivotal role of a handful of lncRNAs during cardiac regeneration. For example, MALAT1 lncRNA is upregulated during MI. Conversely, the downregulation of MALAT1 regulates the mitochondrial activity of endothelial cells and the cell viability of cardiac progenitor cells [217,218]. Furthermore, macrophage-M1-derived EVs carrying MALAT1 also have an impact on endothelial cell viability [193]. In addition, in vitro experiments have also reported that MALAT1 can influence CF proliferation and collagen deposition [113]. Interestingly, the reported mechanisms of action mainly act as sponges of miRNAs, i.e., miR-26 and miR-25-3p in the case of microvascular endothelial cells, miR-125 in cardiac progenitor cells, and miR-145 in fibroblasts [113,193,217,218]. Importantly, the modulation of such miRNAs leads to the deregulation of key components of mitochondrial activity such as Mfn1 (mitofusin-1) by miR-26, proliferation modulators such as CDC42 (cell division control protein 42 homolog) by miR-25-3p, epigenetic regulators such as Jmj6b by miR-125, or fibrotic inductors such as TGF-β1 [113,193,217,218].

Discordant evidence has been provided for H19 lncRNA in MI. High levels of H19 have been detected in the plasma of MI patients [235], in a neonatal MI model [222], and in an in vitro model of hypoxia/reperfusion CMs [221]. However, this lncRNA was downregulated in a mouse model of MI [219]. Importantly, forced H19 expression, which regulates miR-22-3p in CMs targeting Kdm3a (lysine demethylase 3A), reduces infarct size and improves cardiac fibrosis and thus cardiac performance. Additional evidence regarding H19 was provided by Li et al. [220] in cardiac progenitor cells after hypoxia, for which it was reported that such downregulation decreases the proliferation and migration of cardiac progenitor cells, a process mediated by miR-200a-3p and Sirt1 regulation.

Mechanistically, Luo et al. [221] related that H19 downregulation leads to increased cell viability and reduced apoptosis, inflammatory release, and oxidative stress, in part by regulating miR-675 and thus PPARα, in both in vitro and in vivo models of hypoxia/reperfusion in CMs. Furthermore, Choong et al. [222] reported that H19 overexpression in a mouse heart leads to cardiac dilation and fibrosis, whereas H19 genetic ablation significantly reduces post-MI remodeling by interacting with YB-1 and thus modulating collagen expression and fibrosis [222]. While these data clearly demonstrate the pivotal role of H19 in MI, supporting its functional role in different cell types and molecular cascades, the discordant evidence remains to be elucidated.

The role of MIAT lncRNA in MI has been assessed in MIAT deficient mice. TAC surgical procedures in MIAT-deficient mice reduced heart/body ratios and decreased CM cross-sectional area and apoptosis as well as levels of cardiac fibrosis [236]. In addition, inflammatory factors are significantly reduced by the silencing of MIAT throughout the activation of PI3K/Akt signaling pathway [223]. Additional evidence in this vein was reported in the study by Tan et al. [41], in which a MIAT knockdown led to the reversal of cell proliferation, apoptosis, and inflammatory injury in miR-181a-5p-silenced or JAK2-overexpressing OGD-included CMs; further evidence was provided by Dong et al. [224], who reported that MIAT sponges miR-182-5p and thereby regulates GPRC5A expression in vitro. Importantly, MIAT knockdown reduces myocardial injury caused by I/R treatment in vivo [41].

TUG1 lncRNA expression increases after MI. TUG1 knockdown inhibits Ang-II-induced cardiac myofibroblast activation, acting as a sponge of miR-133b, which, in turn, modulates CTGF expression and, therefore, myofibroblast activation [118]. Additionally, Sun et al. [225] also reported that TUG1 knockdown suppresses cell viability and migration and improves the collagen production of TGF-β1-treated CFs by sponging miR-590 and thus regulating FGF1.

Besides those lncRNAs with widely reported influences on several aspects of cardiac regeneration, the reported degree of involvement of additional lncRNAs is currently limited, for which participation occurs through two distinct mechanisms. First, there are those directly interacting with distinct proteins, as revealed by RNA–protein pull-down assays, such as Sngh1/PTEN acting on PI3K/AKT signaling, lncDACH1/PPIA modulating YAP1 signaling [227], Nppa-AS1/SFPQ, CPR/Mcm3, Sarrah/Nfr2, and Airn/Igf2bp2-Rap1 [226,228,229,230,231]. Such regulatory mechanisms impact CM proliferation, as Ponnusamy et al. [229] reported for CPR. Second, two distinct lncRNAs, namely, Carel and AZIN2-sv, modulate CM replication and proliferation by sponging miR-216 and miR-214, thus regulating Trp53inp1/Itm2a and PTEN/PI3k/AKT signaling, respectively [232,234]. In addition, AZIN2-sv can also influence angiogenesis by directly associating with Tln1 and ITFGb1 [234]. Overall, these data highlight the emerging functional role of lncRNAs during cardiac regeneration, demonstrating complex regulatory mechanisms that can influence distinct signaling pathways and thus biological responses.

## 7. circRNAs in Cardiac Infarction and Regeneration

Circular RNAs (circRNAs) comprise a large class of non-coding RNAs that are produced by a non-canonical splicing event called back splicing. During this process, a downstream splice-donor site is covalently linked to an upstream splice-acceptor site. Viroids were the first circRNA molecules to be discovered and, more recently, they have also been identified in eukaryotic cell lines. CircRNAs display differential expression across species, developmental stages, and pathologies. In addition, their lack of free ends confers increased stability when compared with linear transcripts, rendering them good candidate biomarkers. In this context, increasing evidence has been reported for circRNAs in the pathogenesis of multiple cardiovascular diseases, including cardiac injury and regeneration, as detailed in the following paragraphs.

### 7.1. The Role of circRNAs in Cardiac Inflammation

Within the context of inflammation, several studies have provided convincing evidence of the differential levels of contribution of circRNAs to cardiac inflammation during MI. Several of these circRNAs display increased plasma expression levels, while others are downregulated. Furthermore, experimental evidence obtained using both in vitro and in vivo systems has demonstrated that in most cases, these circulating circRNAs act as microRNA sponges that subsequently modulate the expression of key signaling pathways that impact the inflammatory response or even directly modulate inflammatory markers. In the following paragraphs, we summarize the current knowledge of the functional roles of the circRNAs that affect cardiac inflammation (Table 2).

Several circRNAs are upregulated in AMI patients, such as circITGB1 and circ_0023461, while studies have shown that circHelz and circ_0007059 are elevated in MI in mice [81,237,238,239]. Additionally, numerous other circRNAs are upregulated in experimental hypoxia models in vitro, such as circ_0023461, circHelz, circ_0007059, circ_0001747, circTRRAP, and circMAT2B [80,81,238,239,242].

CircITGB1 competitively binds to miR-342-3p and inhibits its expression, resulting in the increased expression of NFAT-activating molecule 1 (NFAM1). circITGB1 controls dendritic cell maturation by targeting miR-342-3p and NFAM1. circITGB1 also exacerbated cardiac damage and regulated miR-342-3p and NFAM1 expression in a mouse AMI model [237]. Similarly, circ_0023461 expression is upregulated in AMI patients and hypoxia-induced AC16 cells, and it can upregulate PDE4D expression by acting as a molecular sponge for miR-370-3p in AC16 cells [238].

In mice, the overexpression of circHelz causes CM injury in neonatal ventricular CMs by activating the NLRP3 inflammasome and inducing pyroptosis via inhibiting miR-133a-3p function [81].

Circ_0007059 expression was elevated and miR-378 and miR-383 expression were downregulated in H_2_O_2_-treated mouse CMs and the infarcted hearts of an MI mouse model. Circ_0007059 knockdown improves CM viability, suppresses apoptosis after H_2_O_2_ treatment, and represses H_2_O_2_-induced inflammation, as assessed by IL1β, IL18, and CCL5 expression. Importantly, circ_0007059 acts as a miR-378 and miR-383 sponge. These data suggest that circ_0007059 expression is upregulated in mouse CMs, in response to oxidative stress, and in the cardiac tissues of an MI mouse model, supporting its involvement in the pathogenesis of MI by targeting miR-378 and miR-383 [239].

Circ_0001747 directly targets miR-199b-3p in HL-1 cells. miR-199b-3p overexpression partly overturns exosomal circ_0001747-mediated protective effects in hypoxia/reperfusion-induced HL-1 cells. miR-199b-3p silencing alleviates hypoxia/reperfusion-induced damage in HL-1 cells partly by upregulating MCL1. Overall, these results indicate that adipose-derived-stem-cell (ADSC)-derived exosomes with high amounts of circ_0001747 attenuate hypoxia/reperfusion-induced HL-1 dysfunction partly by targeting miR-199b-3p/MCL1 signaling [242].

Additionally, Zhang et al. [240] demonstrated that an experimental downregulation of circTRRAP promotes cell growth but inhibits apoptosis, inflammation, and oxidative stress in hypoxic cells. The regulatory effects of circTRRAP on the hypoxic cells were associated with the sponge function of miR-370-3p. In addition, PAWR serves as the target for miR-370-3p, and it is regulated by circTRRAP/miR-370-3p axis. Thus, a protective role of miR-370-3p was achieved by downregulating the PAWR expression in the hypoxia-treated AC16 cells [240].

CircMAT2B expression was notably upregulated in oxygen-glucose-deprivation (OGD)-induced H9c2 cells. Moreover, circMAT2B knockdown effectively decreased OGD-induced apoptosis, ROS generation, and the expression of IL-1β, IL-6, and TNF-α. Furthermore, it was determined that miR-133 is positively regulated by si-circMAT2B. CircMAT2B knockdown attenuates OGD-induced H9c2 cell damage and alleviates the OGD-induced inhibition of PI3K/AKT and Raf/MEK/ERK pathways through the upregulation of miR-133. In brief, circMAT2B knockdown operates as an inflammatory inhibitor in OGD-induced H9c2 cells through the upregulation of miR-133 [80].

Decreased expression of circRNAs in AMI patients and mice has also been reported, specifically in reference to circUBXN7 [241]. CircUBXN7 was downregulated in patients and mice with AMI as well as in hypoxia/reperfusion-treated cells. Overexpression of circUBXN7 mitigates hypoxia/reperfusion-mediated apoptosis and the secretion of inflammatory factors including IL-6, TNF-α, and IL-1β. In addition, circUBXN7 suppresses cell apoptosis and inflammatory reactions induced by hypoxia/reperfusion via targeting miR-622. miR-622 targets MCL1 to restrain its expression in H9c2 cells. The knockdown of MCL1 abrogates the circUBXN7-mediated alleviation of apoptosis and inflammation after hypoxia/reperfusion treatment [241].

### 7.2. The Role of circRNAs in Cardiac Fibrosis

Within the context of cardiac fibrosis, additional supporting evidence on the functional role of circRNAs is also emerging, including with respect to several circRNAs with increased expression after MI, such as circPAN, or decreased expression, such as circ_LAS1L and circNFIB, while in other cases only in vitro evidence of their functional role has been reported (i.e., circ_0005019) [243,244,245,246]. Additionally, three distinct circRNAs can also influence cardiac fibrosis in vivo: circCELF1, circRNA 010567, and circUbe3a [247,248,258].

CircPAN3 was upregulated in a rat MI model. Importantly, circPAN3 knockdown attenuates cardiac fibrosis after MI and blunts in vitro cell proliferation and migration mediated by TGFβ1 administration. miR-221 is a target involved in circPAN3-mediated cardiac fibrosis after MI, wherein it negatively regulates FoxO3 and thus causes ATG7 transcription inhibition. Such molecular mechanisms were further validated in vivo [243].

Circ_LAS1L is downregulated in acute MI patients and CFs. In addition, it can directly bind to miR-125b. It thus promotes the expression of downstream target genes such as SFRP5, ultimately inhibiting the activation, proliferation, and migration of CFs in vitro and promoting apoptosis [244]. However, it remains to be elucidated if such effects are also observed in vivo.

Zhu et al. [245] reported that circNFIB was decreased in post-MI murine heart samples and in primary adult CFs treated with TGF-β. Moreover, circNFIB overexpression decreased fibroblast cell proliferation in vitro and sponged miR-433 [245]. While these data support a plausible role of circNFIB in cardiac fibrosis, no evidence obtained in vivo supports such a role.

In CFs, circ_0005019 presents inhibitory effects on cell proliferation and migration. Circ_0005019 acts as a miR-499-5p sponge, regulating the expression of its target gene Kcnn3 [246]. However, the molecular mechanisms that directly affect cell proliferation and migration remain largely elusive.

CircCELF1 enhanced DKK2 expression by sponging miR-636, thereby inhibiting myocardial fibrosis progression in a mouse model of acute MI, and circRNA010567 inhibition improved cardiac function and alleviated myocardial fibrosis and apoptosis in a rat model of acute MI, constituting processes that seem to be mediated by the inhibition of the TGF-β1 signaling pathway [247,258].

On the other hand, circUbe3a, which was loaded into M2-derived small EVs, led to functional changes in CFs during progressive M2 macrophage infiltration after MI. The manipulation of circUbe3a in small EVs generating conditions conducive to the silencing or overexpression of circUbe3a altered the proliferation, migration, and phenotypic transformation of CFs in vitro [248]. Such effects are provided, in part, by sponging miR-138-5p and, consequently, repressing RhoC. In vivo, the administration of M2-derived SEVs or the overexpression of circUbe3a significantly increases myocardial fibrosis after acute MI [248]. In summary, these data reveal the increasingly prominent and complex role of circRNA’s gene-regulatory networks impacting cardiac fibrosis.

### 7.3. The Role of circRNAs in Other Biological Processes of Cardiac Infarction

While circRNAs can also influence several other biological processes that concomitantly occur during cardiac infarction besides inflammation and fibrosis, such as autophagy, apoptosis, and oxidative stress signaling, the evidence in this regard is still limited. Among those circRNAs currently reported to play such roles are circFoxo3 and circSLC8A1 [249,250].

CircFoxo3 was downregulated in a rat model of MI, and circFoxo3 overexpression ameliorated MI-induced cardiac dysfunction and reduced MI-induced autophagy. Mechanistically, circFoxo3 gain-of-function represses oxygen-glucose-deprivation (OGD)-induced autophagy, apoptosis, inflammation, and injury of CM in vitro. In addition, it relieves myocardial ischemia/reperfusion injury by suppressing autophagy via the inhibition of HMGB1 (high mobility group box 1) by repressing KAT7 (lysine acetyltransferase 7) in MI [249].

Exosomal circSLC8A1 exacerbates the hypoxia-induced repression of cell viability but promotes cell apoptosis, inflammation, and oxidative stress. The knockdown of circSLC8A1 ameliorates hypoxia-mediated cell injury. circSLC8A1 directly targets miR-214-5p, and miR-214-5p downregulation reverts the effects of si-circSLC8A1 on hypoxia-treated CMs. TEAD1 (TEA domain transcription factor 1), a target of miR-214-5p, is thusly upregulated. These results suggest that circSLC8A1 aggravates cell damage in hypoxia-treated CMs through the regulation of TEAD1 via sponging miR-214-5p [250].

### 7.4. The Role of circRNAs in Cardiac Regeneration

Increasing evidence is being reported regarding the functional roles of circRNAs during cardiac regeneration. Four distinct cirRNAs have been recently reported: circNfix, circHFIB, circCDYL, and circMdc1 [245,251,252,253].

CircNfix is expressed in the adult hearts of mice, rats, and humans. CircNfix downregulation increases CM proliferation and angiogenesis while inhibiting CM apoptosis following MI in mice, enhancing cardiac function and thus improving prognosis. At the molecular level, circNfix enhances Nedd4l (NEDD4-like E3 ubiquitin protein ligase) and Ybx1 (Y-box binding protein 1) interaction, inducing Ybx1 degradation through ubiquitination while repressing cyclin A2 and cyclin B1 expression. Furthermore, circNfix sponges miR-214 and thus promotes Gsk3β (glycogen synthase kinase 3 β) expression, thereby decreasing β-catenin activity [251].

On the other hand, circCDYL is downregulated in myocardial tissues and hypoxic myocardial cells in vitro. Importantly, gain- and loss-of-function assays of circCDYL performed using a mouse model of acute MI improved and aggravated heart function, respectively. Furthermore, circCDYL promoted CM proliferation in vitro. Molecularly, circCDYL can sponge miR-4793-5p and thus regulate its expression, thereby influencing miR-4793-5p-targeted APP expression [252]. However, how circCDYL acts in vivo remains to be established.

CircMdc1 levels are upregulated in the postnatal mouse heart, while they are downregulated during neonatal cardiac regeneration. Forced circMdc1 expression inhibits CM proliferation, while silencing promotes CM cell cycle re-entry in vitro. In vivo, circMdc1 knockdown improves cardiac regeneration and heart repair accompanied by enhanced heart function. Importantly, circMdc1 can block Mdc1 translation by binding to PABP, leading to DNA damage and CM chromosome instability [253]. Additional efforts are required to fully elucidate this circRNA’s function in vivo in the context of cardiac regeneration.

Additionally, two circRNAs have been reported to play functional roles during cardiac regeneration and cardiac inflammation (circSamd4) and fibrosis (circHipk3), respectively [254,255,256,257].

In vivo, Zheng et al. [254] demonstrated that circSamd4 overexpression induces CM proliferation while preventing apoptosis by modulating oxidative stress generation and mitochondrial dynamics. In turn, a reduced size of the fibrotic area and improved cardiac function after MI was observed. On the other hand, Hu et al. [255] reported that the inhibition of circSAMD4A expression reduced hypoxia/reperfusion-induced apoptosis and inflammation by inhibiting the expression of miR-138-5p. Thus, these data suggest that circSAMD4 might have distinct functional roles, namely, a protective role during acute MI and a negative role during hypoxia/reperfusion.

CircHipk3 overexpression attenuates cardiac dysfunction and decreases fibrotic area after MI in mice by modulating Notch signaling and sponging miR-133. Importantly, circHipk3 overexpression promotes coronary vessel endothelial cell proliferation, migration, and tube-forming capacity and subsequent angiogenesis [256]. Deng et al. [257] provided additional evidence for the functional role of circHipk3, demonstrating that circHipk3 affects the cytoplasmic calcium concentration by modulating miR-17-3p and thus Adcy6. In vivo experiments showed that the downregulation of circHipk3 can alleviate fibrosis and maintain cardiac function in post-MI mice. Therefore, it seems that either the overexpression or downregulation of circHipk3 may improve cardiac function and decrease fibrotic scar generation in mice. Additional experiments are required to reconcile such findings.

## 8. Conclusions and Perspectives

Cardiovascular diseases represent an enormous socioeconomic burden worldwide and are the leading cause of mortality despite the increasing and significant efforts undertaken to improve treatments [259,260]. In this context, MI is a major public health issue with increasing prevalence [261,262]. Over the last decade, our understanding of the molecular mechanisms that drive MI has greatly advanced in terms of searching for potential therapeutic strategies to heal the damaged heart. The discovery that several species can achieve complete cardiac regeneration, such as the axolotl and zebrafish, has provided experimental models with which to faithfully test those therapeutic strategies, including therein mammals with a temporal window of effective cardiac regeneration [22,263,264]. In the last few years, the research on the molecular mechanisms that drive cardiac injury and regeneration has provided ample evidence that non-coding RNAs also play essential roles in these biological processes. Within this review, we have provided a state-of-the-art summary of the current contributions of miRNAs, lncRNAs, and circRNAs to distinct biological processes such as inflammation, fibrosis, apoptosis, and cell proliferation, which are key elements of cardiac injury and regeneration. It is important to highlight the continuous emergence of evidence relating the cross-talk between coding and non-coding RNAs, as well as among non-coding RNAs (i.e., microRNA, lncRNAs and circRNAs) impacting key signaling pathways leading to inflammation, such as NF-kB and PI3K/AKT, or fibrosis, such as Tgfβ1/Smads, as depicted in Figure 1 and Figure 2, respectively. Over the next coming years, we will witness the further elucidation of the functional roles of these non-coding RNAs in cardiac injury and regeneration. In the meantime, we will deepen our understanding of the complex regulatory networks intermingling non-coding- and protein-signaling pathways, thus paving the way for the design of novel therapeutic tools with which to heal dysfunctional hearts at the bedside.

## Figures and Tables

**Table 1:** List of lncRNAs and their corresponding sponged microRNAs affecting distinct proteins and signaling pathways. Highlighted ncRNAs and proteins (in bold) correspond to those lncRNAs and proteins with which they have been reported to physically interact, i.e., by means of pull-down assays.

Cardiac Inflammation					
lncRNA	miRNA	Protein	Signaling	Functional Role	Ref.
KCNQ1OT1	miR-130	ZNF91	-		[83]
LUCAT1	miR-181	-	-	ROS, inflammation, apoptosis	[40]
HULC	miR-29	-	-	Inflammation, angiogenesis	[194]
HULC	miR-377	NLRP3/Caspase-1/IL-1β	-	Inflammation, apoptosis	[195]
TTTY15	miR-98	CRP	-	Myocardial injury	[192]
MIAT2	miR-15	-	-	Inflammation	[196]
MIRT2	miR-377	-	-		[197]
GM2691	-	-	Akt	Inflammation	[198]
MIRT1	-	-	NF-kB	Cardiomyocytes, inflammation	[199]
NEAT1	-	-	-	Myocardial inflammation	[200]
LUNAR1	-	-		Inflammation	[201]
SNHG8	-	-	-	Inflammation	[202]
**Cardiac Fibrosis**					
**lncRNA**	**miRNA**	**Protein**	**Signaling**	**Functional Role**	**Ref.**
**SAFE**	-	**Sfrp2, HuR**	-	Fibroblasts, fibrosis	[203]
**WISPER**	-	**Tia1-related protein**	Collagen	Proliferation, fibrosis	[204]
**SAIL**	-	**Safb**	Collagen	Fibrosis	[205]
**CFAST**	-	**Colt1, Trap1**	-	Fibrosis	[206]
PCFL	miR-378	Grb2	Collagen	Fibrosis	[207]
NORAD	miR-577	Cobll1	Collagen	Fibrosis	[208]
PFL	Let-7d	Ptafr	-	Fibrosis	[209]
MHRT	miR-3185	-	Collagen	Fibrosis	[210]
n379519	miR-30	-	Collagen	Fibrosis	[147]
XIST	miR-155	-	-	Fibrosis	[121]
lncRNA554	-	-	TGF-β1	Fibrosis	[211]
lnc-ANG362	-	Smad7	-	Fibrosis	[212]
lncR-30245	-	-	TGF-β1, Pparg	Fibrosis	[213]
N1LR	-	TGF-β1, Smads	-	Fibrosis, Inflammation	[214]
FGD5-AS1	miR-223	-	Akt	Cardiac fibrosis, inflammation, apoptosis	[91]
**Other Biological Processes Related to Cardiac Infarction**					
**lncRNA**	**miRNA**	**Protein**	**Signaling**	**Functional Role**	**Ref.**
MIRT1	-	-	NF-kβ	Fibroblasts, apoptosis, ROS, inflammation	[215]
GAS5	miR-21	-	-	Myocardial apoptosis	[178]
LNC_000898	miR-375	Pdk1	-	Cardiac apoptosis	[216]
**Cardiac regeneration**					
**lncRNA**	**miRNA**	**Protein**	**Signaling**	**Functional Role**	**Ref.**
MALAT1	miR-26	Mfn1	-	Mitochondrial activity	[217]
MALAT1	miR-25-3p	CDC42	-	-	[193]
MALAT1	miR-125	Jmj6b	-	Endothelial cell viability	[218]
MALAT1	miR-145	TGF-β1	-	Fibroblast proliferation	[113]
H19	miR-22-3p	Kdm3a	-	Fibrosis	[219]
H19	miR-200a-3p	Sirt1	-	Cardiac progenitor proliferation	[220]
H19	miR-675	PPar	-	Cell viability, apoptosis, ROS, inflammation	[221]
**H19**	-	**YB-1**	-	Fibrosis	[222]
MIAT	-	-	PI3K/AKT	-	[223]
MIAT	miR-181a-5p	JAK2	-	Cell proliferation, apoptosis, inflammation	[41]
MIAT	miR-182-5p	GPRC5A	-	-	[224]
TUG1	miR-133b	-	-	-	[118]
TUG1	miR-590	Fgf1	TGF-β	Fibrosis	[225]
**SNHG1**	-	**PTEN**	PI3K/AKT	-	[226]
**lncDACH1**	-	**PPQA**	YAP1	-	[227]
**NPPA-AS1**	-	**SFPQ**	-	-	[228]
**CPR**	-	**Mcm3**	-	Cardiomyocyte proliferation, scar formation	[229]
SARRAH	-	Nrf2	-	-	[230]
**AIM**	-	**Igf2bp2, Rap1**	-	-	[231]
CAREL	miR-296	Trp53inp1, Itm2a	-	Cardiomyocyte replication	[232]
AZIN2-sv	miR-214	PTEN	PI3K/AKT	Cardiomyocyte proliferation	[233]
**AZIN2-sv**	-	**Tln1, ITGB1**	-	Angiogenesis	[234]

**Table 2:** List of circRNAs and their corresponding sponged microRNAs that, accordingly, affect the expression of distinct proteins and signaling pathways. circRNAs and proteins that are highlighted (in bold) correspond to those circRNAs and proteins that have been reported to physically interact with each other, i.e., by means of pull-down assays.

Cardiac Inflammation				
circRNA	miRNA	Protein	Signaling	Ref.
circITGB1	miR-342-3p	NFAM1	-	[237]
circ_0023461	miR-370-3p	PDE4D	-	[238]
circHelz	miR-133a-3p	NLRP3	-	[81]
circ_0007059	miR-378, miR-383	-	-	[239]
circTRRAP	miR-370-3p	PAWR	-	[240]
circMAT2B	miR-133	-	PI3K/AKT, Raf/MEK/ERK	[80]
circUBXN7	miR-622	MCL1	-	[241]
circ_0001747	miR-199-3p	MCL1	-	[242]
**Cardiac Fibrosis**				
**lncRNA**	**miRNA**	**Protein**	**Signaling**	**Ref.**
circPAN	miR-221	Foxo3	TGF-β	[243]
circ_LAS1L	miR-125b	SFRP5	-	[244]
circNFIB	miR-433	-	TGF-β	[245]
circ_0005019	miR-499-5p	Kcnn3	-	[246]
circCELF1	miR-636	Dkk2	TGF-β	[247]
circUbe3a	miR-138-5p	-	-	[248]
**Other Biological Processes Related to Cardiac Infarction**				
**lncRNA**	**miRNA**	**Protein**	**Signaling**	**Ref.**
circFoxo3	-	KAT7	-	[249]
circSLC8A1	miR-214	TEAD4	-	[250]
**Cardiac Regeneration**				
**lncRNA**	**miRNA**	**Protein**	**Signaling**	**Ref.**
**circNfix**	-	**Neddl4, Ybx1**	-	[251]
circCDYL	miR-4793-5p	APP	-	[252]
**circMdc1**	-	**PABP/MDC1**	-	[253]
circSmad4	-	-	-	[254]
circSmad4	miR-138-5p	-	-	[255]
circHipk3	miR-133	-	-	[256]
cirHipk3	miR-17-3p	-	-	[257]

## Data Availability

Not applicable.

## References

[1] Bhatt A.S., Ambrosy A.P., Velazquez E.J. (2017). Adverse Remodeling and Reverse Remodeling After Myocardial Infarction. Curr. Cardiol. Rep..

[2] Thygesen K., Alpert J.S., Jaffe A.S., Chaitman B.R., Bax J.J., Morrow D.A., White H.D. (2018). Fourth Universal Definition of Myocardial Infarction. J. Am. Coll. Cardiol..

[3] Basara G., Bahcecioglu G., Ozcebe S.G., Ellis B.W., Ronan G., Zorlutuna P. (2022). Myocardial infarction from a tissue engineering and regenerative medicine point of view: A comprehensive review on models and treatments Myocardial infarction from a tissue engineering and regenerative medicine point of view: A comprehensive review on m. Biophys. Rev..

[4] Prabhu S.D., Frangogiannis N.G., Service M., Einstein A. (2016). The Biological Basis for Cardiac Repair After Myocardial Infarction: From Inflammation to Fibrosis. Circ. Res..

[5] Frangogiannis N.G. (2015). Pathophysiology of Myocardial Infarction. Compr. Physiol..

[6] McCoy C.E., Sheedy F.J., Qualls J.E., Doyle S.L., Quinn S.R., Murray P.J., O’Neill L.A.J. (2010). IL-10 inhibits miR-155 induction by toll-like receptors. J. Biol. Chem..

[7] Chen Q., Lv J., Yang W., Xu B., Wang Z., Yu Z., Wu J., Yang Y., Han Y. (2019). Targeted inhibition of STAT3 as a potential treatment strategy for atherosclerosis. Theranostics.

[8] Kyoko I.O., Taisuke K., Minako I., Yoshimura A. (2013). SOCS, inflammation, and cancer. JAKSTAT.

[9] Zhu1 M., Goetsch S.C., Wang Z., Luo R., Hill J.A., Schneider J., Morris S.M., Liu Z.-P. (2015). FoxO4 Promotes Early Inflammatory Response Upon Myocardial Infarction via Endothelial Arg1. Circ. Res..

[10] Mouton A.J., Rivera O.J., Lindsey M.L. (2018). Myocardial infarction remodeling that progresses to heart failure: A signaling misunderstanding. Am. J. Physiol. Circ. Physiol..

[11] Yang J., Zhang L., Yu C., Yang X.F., Wang H. (2014). Monocyte and macrophage differentiation: Circulation inflammatory monocyte as biomarker for inflammatory diseases. Biomark. Res..

[12] Slava E., Lavine K.J., Randolph G.J. (2014). Origin and Functions of Tissue Macrophages. Immunity.

[13] Epelman S., Lavine K.J., Beaudin A.E., Sojka D.K., Carrero J.A., Calderon B., Brija T., Gautier E.L., Ivanov S., Ansuman T. (2014). Embryonic and adult-derived resident cardiac macrophages are maintained through distinct mechanisms at steady state and during inflammation. Immunity.

[14] Skrzeczyńska-Moncznik J., Bzowska M., Loseke S., Grage-Griebenow E., Zembala M., Pryjma J. (2008). Peripheral blood CD14high CD16+ monocytes are main producers of IL-10. Scand. J. Immunol..

[15] Mukherjee R., Kanti Barman P., Kumar Thatoi P., Tripathy R., Kumar Das B., Ravindran B. (2015). Non-Classical monocytes display inflammatory features: Validation in Sepsis and Systemic Lupus Erythematous. Sci. Rep..

[16] Eigsti R.L., Sudan B., Wilson M.E., Graff J.W. (2014). Regulation of activation-associated microRNA accumulation rates during monocyte-to-macrophage differentiation. J. Biol. Chem..

[17] Martinez F.O., Gordon S. (2014). The M1 and M2 paradigm of macrophage activation: Time for reassessment. F1000Prime Rep..

[18] Wang C., Zhang C., Liu L., Xi A., Chen B., Li Y., Du J. (2017). Macrophage-Derived mir-155-Containing Exosomes Suppress Fibroblast Proliferation and Promote Fibroblast Inflammation during Cardiac Injury. Mol Ther..

[19] Yonggang M., Alan J.M., Merry L.L. (2018). Cardiac macrophage biology in the steady-state heart, the aging heart, and following myocardial infarction. Transl. Res..

[20] Nikolaos G., Frangogiannis M. (2015). Inflammation in cardiac injury, repair and regeneration Nikolaos. Curr. Opin. Cardiol..

[21] Hu Y., Zhang H., Lu Y., Bai H., Xu Y., Zhu X., Zhou R., Ben J., Xu Y., Chen Q. (2011). Class A scavenger receptor attenuates myocardial infarction-induced cardiomyocyte necrosis through suppressing M1 macrophage subset polarization. Basic Res. Cardiol..

[22] Frangogiannis N.G. (2014). The inflammatory response in myocardial injury, repair, and remodelling. Nat. Rev. Cardiol..

[23] Begeman I.J., Kang J. (2018). Transcriptional programs and regeneration enhancers underlying heart regeneration. J. Cardiovasc. Dev. Dis..

[24] Quaife-Ryan G.A., Sim C.B., Ziemann M., Kaspi A., Rafehi H., Ramialison M., El-Osta A., Hudson J.E., Porrello E.R. (2017). Multicellular transcriptional analysis of mammalian heart regeneration. Circulation.

[25] Wei K., Serpooshan V., Hurtado C., Diez-Cuñado M., Zhao M., Maruyama S., Zhu W., Fajardo G., Noseda M., Nakamura K. (2015). Epicardial FSTL1 reconstitution regenerates the adult mammalian heart. Nature.

[26] Gemberling M., Karra R., Dickson A.L., Poss K.D. (2015). Nrg1 is an injury-induced cardiomyocyte mitogen for the endogenous heart regeneration program in zebrafish. Elife.

[27] Marinescu M.C., Lazar A.L., Marta M.M., Cozma A., Catana C.S. (2022). Non-Coding RNAs: Prevention, Diagnosis, and Treatment in Myocardial Ischemia–Reperfusion Injury. Int. J. Mol. Sci..

[28] Garcia-padilla C., Lozano-velasco E., Garcia-lopez V., Aranega A., Franco D., Garcia-martinez V., Lopez-sanchez C. (2022). Comparative Analysis of Non-Coding RNA Transcriptomics in Heart Failure. Biomedicines.

[29] Friedman R.C., Farh K.K.H., Burge C.B., Bartel D.P. (2009). Most mammalian mRNAs are conserved targets of microRNAs. Genome Res..

[30] Mendell J.T., Olson E.N. (2012). MicroRNAs in stress signaling and human disease Joshua. Cell.

[31] Fiedler J., Thum T. (2013). MicroRNAs in myocardial infarction. Arter. Thromb. Vasc. Biol..

[32] Varzideh F., Kansakar U., Donkor K., Wilson S., Jankauskas S.S., Mone P., Wang X., Lombardi A., Santulli G. (2022). Cardiac Remodeling After Myocardial Infarction: Functional Contribution of microRNAs to Inflammation and Fibrosis. Front. Cardiovasc. Med..

[33] Collins L., Binder P., Chen H., Wang X. (2020). Regulation of Long Non-coding RNAs and MicroRNAs in Heart Disease: Insight Into Mechanisms and Therapeutic Approaches. Front. Physiol..

[34] Meng Z., Chen C., Cao H., Wang J., Shen E. (2019). Whole transcriptome sequencing reveals biologically significant RNA markers and related regulating biological pathways in cardiomyocyte hypertrophy induced by high glucose. J. Cell. Biochem..

[35] Altesha M.A., Ni T., Khan A., Liu K., Zheng X. (2019). Circular RNA in cardiovascular disease. J. Cell. Physiol..

[36] Wen Z.J., Xin H., Wang Y.C., Liu H.W., Gao Y.Y., Zhang Y.F. (2021). Emerging roles of circRNAs in the pathological process of myocardial infarction. Mol. Ther. Nucleic Acids.

[37] Wang C., Jing Q. (2018). Non-coding RNAs as biomarkers for acute myocardial infarction review-article. Acta Pharmacol. Sin..

[38] Liu H.Y., Yu L.F., Zhou T.G., Wang Y.D., Sun D.H., Chen H.R., Hou Y.F. (2020). Lipopolysaccharide-stimulated bone marrow mesenchymal stem cells-derived exosomes inhibit H2O2-induced cardiomyocyte inflammation and oxidative stress via regulating miR-181a-5p/ATF2 axis. Eur. Rev. Med. Pharmacol. Sci..

[39] Wei Z., Qiao S., Zhao J., Yihai L., Qiaoling L., Zhonghai W., Qing D., Lina K., Biao X. (2019). miRNA-181a over-expression in mesenchymal stem cell-derived exosomes influenced inflammatory response after myocardial ischemia-reperfusion injury. Life Sci..

[40] Xiao S.H., Wang Y., Cao X., Su Z. (2021). Long non-coding RNA LUCAT1 inhibits myocardial oxidative stress and apoptosis after myocardial infarction via targeting microRNA-181a-5p. Bioengineered.

[41] Tan J.K., Ma X.F., Wang G.N., Jiang C.R., Gong H.Q., Liu H. (2021). LncRNA MIAT knockdown alleviates oxygen-glucose deprivation-induced cardiomyocyte injury by regulating JAK2/STAT3 pathway via miR-181a-5p. J. Cardiol..

[42] Cao R.Y., Li Q., Miao Y., Zhang Y., Yuan W., Fan L., Liu G., Mi Q., Yang J. (2016). The emerging role of MicroRNA-155 in cardiovascular diseases. BioMed Res. Int..

[43] O’Connell R.M., Taganov K.D., Boldin M.P., Cheng G., Baltimore D. (2007). MicroRNA-155 is induced during the macrophage inflammatory response. Proc. Natl. Acad. Sci. USA.

[44] Suzuki H.I., Arase M., Matsuyama H., Choi Y.L., Ueno T., Mano H., Sugimoto K., Miyazono K. (2011). MCPIP1 ribonuclease antagonizes dicer and terminates microRNA biogenesis through precursor microRNA degradation. Mol. Cell..

[45] Niu J., Jin Z., Kim H., Kolattukudy P.E. (2015). MCP-1-induced protein attenuates post-infarct cardiac remodeling and dysfunction through mitigating NF-κB activation and suppressing inflammation-associated microRNA expression. Basic Res. Cardiol..

[46] Agudelo J.S.H., Braga T.T., Amano M.T., Cenedeze M.A., Cavinato R.A., Peixoto-Santos A.R., Muscará M.N., Teixeira S.A., Cruz M.C., Castoldi A. (2017). Mesenchymal stromal cell-derived microvesicles regulate an internal pro-inflammatory program in activated macrophages. Front. Immunol..

[47] Essandoh K., Li Y., Huo J., Fan G.C. (2016). MiRNA-mediated macrophage polarization and its potential role in the regulation of inflammatory response. Shock.

[48] Li Y., Zhang C., Wu Y., Han Y., Cui W., Jia L., Cai L., Cheng J., Li H., Du J. (2012). Interleukin-12p35 deletion promotes CD4 T-cell-dependent macrophage differentiation and enhances angiotensin II-Induced cardiac fibrosis. Arter. Thromb. Vasc. Biol..

[49] Song M., Cui X., Zhang J., Li Y., Li J., Zang Y., Li Q., Yang Q., Chen Y., Cai W. (2022). Shenlian extract attenuates myocardial ischaemia-reperfusion injury via inhibiting M1 macrophage polarization by silencing miR-155. Pharm. Biol..

[50] Matsushima S., Tsutsui H., Sadoshima J. (2014). Physiological and Pathological Functions of NADPH Oxidases during Myocardial Ischemia-Reperfusion. Trends Cardiovasc. Med..

[51] Ying W., Riopel M., Bandyopadhyay G., Dong Y., Birmingham A., Seo J.B., Ofrecio J.M., Wollam J., Hernandez-Carretero A., Fu W. (2017). Adipose Tissue Macrophage-Derived Exosomal miRNAs Can Modulate in Vivo and in Vitro Insulin Sensitivity. Cell.

[52] Liu S., Chen J., Shi J., Zhou W., Wang L., Fang W., Zhong Y., Chen X., Chen Y., Sabri A. (2020). M1-like macrophage-derived exosomes suppress angiogenesis and exacerbate cardiac dysfunction in a myocardial infarction microenvironment. Basic Res. Cardiol..

[53] Zhang Y., Zhang M., Li X., Tang Z., Wang X., Zhong M., Suo Q., Zhang Y., Lv K. (2016). Silencing MicroRNA-155 Attenuates Cardiac Injury and Dysfunction in Viral Myocarditis via Promotion of M2 Phenotype Polarization of Macrophages. Sci. Rep..

[54] Nazari-Jahantigh M., Wei Y., Noels H., Akhtar S., Zhou Z., Koenen R.R., Heyll K., Gremse F., Kiessling F., Grommes J. (2012). MicroRNA-155 promotes atherosclerosis by repressing Bcl6 in macrophages. J. Clin. Investig..

[55] Zidar N., Boštjančič E., Glavač D., Štajer D. (2011). MicroRNAs, innate immunity and ventricular rupture in human myocardial infarction. Dis. Markers..

[56] He W., Huang H., Xie Q., Wang Z., Fan Y., Kong B., Huang D., Xiao Y. (2016). MIR-155 knockout in fibroblasts improves cardiac remodeling by targeting tumor protein p53-Inducible nuclear protein 1. J. Cardiovasc. Pharmacol. Ther..

[57] Yang L., Wang B., Zhou Q., Wang Y., Liu X., Liu Z., Zhan Z. (2018). MicroRNA-21 prevents excessive inflammation and cardiac dysfunction after myocardial infarction through targeting KBTBD7. Cell Death Dis..

[58] Canfrán-Duque A., Rotllan N., Zhang X., Fernández-Fuertes M., Ramírez-Hidalgo C., Araldi E., Daimiel L., Busto R., Fernández-Hernando C., Suárez Y. (2017). Macrophage deficiency of miR-21 promotes apoptosis, plaque necrosis, and vascular inflammation during atherogenesis. EMBO Mol. Med..

[59] Wang Y., Hou M., Duan S., Zhao Z., Wu X., Chen Y., Yin L. (2022). Macrophage-targeting gene silencing orchestrates myocardial microenvironment remodeling toward the anti-inflammatory treatment of ischemia-reperfusion (IR) injury. Bioact. Mater..

[60] Bejerano T., Etzion S., Elyagon S., Etzion Y., Cohem S. (2018). Nanoparticle Delivery of miRNA-21 mimic to Cardiac Macrophages Improves Myocardial Remodeling after Myocardial Infarction. Nano Lett..

[61] Li Y., Chen X., Jin R., Chen L., Dang M., Cao H., Dong Y., Cai B., Bai G., Justin Gooding J. (2021). Injectable hydrogel with MSNs/microRNA-21-5p delivery enables both immunomodification and enhanced angiogenesis for myocardial infarction therapy in pigs. Sci. Adv..

[62] Poliseno L., Tuccoli A., Mariani L., Evangelista M., Citti L., Woods K., Mercatanti A., Hammond S., Rainaldi G. (2006). MicroRNAs modulate the angiogenic properties of HUVECs. Blood.

[63] Sahoo S., Losordo D.W. (2014). Exosomes and cardiac repair after myocardial infarction. Circ. Res..

[64] de Jong O.G., van Balkom B.W.M., Schiffelers R.M., Bouten C.V.C., Verhaar M.C. (2014). Extracellular vesicles: Potential roles in regenerative medicine. Front. Immunol..

[65] Wei Z., Chen Z., Zhao Y., Fan F., Xiong W., Song S., Yin Y., Hu J., Yang K., Yang L. (2021). Mononuclear phagocyte system blockade using extracellular vesicles modified with CD47 on membrane surface for myocardial infarction reperfusion injury treatment. Biomaterials.

[66] Liu X., Dong Y., Chen S., Zhang G., Zhang M., Gong Y., Li X. (2015). Circulating MicroRNA-146a and MicroRNA-21 Predict Left Ventricular Remodeling after ST-Elevation Myocardial Infarction. Cardiology.

[67] Saba R., Sorensen D.L., Booth S.A. (2014). MicroRNA-146a: A dominant, negative regulator of the innate immune response. Front. Immunol..

[68] Meng Q., Liang C., Hua J., Zhang B., Liu J., Zhang Y., Wei M., Yu X., Xu J., Shi S. (2020). A miR-146a-5p/TRAF6/NF-kB p65 axis regulates pancreatic cancer chemoresistance: Functional validation and clinical significance. Theranostics.

[69] Mukundan L., Bishop G.A., Head K.Z., Zhang L., Wahl L.M., Suttles J. (2005). TNF receptor-assocaited factor 6 is an essential mediator of CD40-Activated Proinflammatory Pathways in Monocytes and Macrophages. J. Immunol..

[70] Chen J., Chen T., Zhou J., Zhao X., Sheng Q., Lv Z. (2021). MiR-146a-5p Mimic Inhibits NLRP3 Inflammasome Downstream Inflammatory Factors and CLIC4 in Neonatal Necrotizing Enterocolitis. Front. Cell Dev. Biol..

[71] Shimada B.K., Yang Y., Zhu J., Wang S., Suen A., Kronstadt S.M., Jeyaram A., Jay S.M., Zou L., Chao W. (2020). Extracellular miR-146a-5p Induces Cardiac Innate Immune Response and Cardiomyocyte Dysfunction. ImmunoHorizons.

[72] Ye E.A., Steinle J.J. (2016). MiR-146a Attenuates Inflammatory Pathways Mediated by TLR4/NF-B and TNF α to Protect Primary Human Retinal Microvascular Endothelial Cells Grown in High Glucose. Mediat. Inflamm..

[73] Chen C., Cai S., Wu M., Wang R., Liu M., Cao G., Dong M., Yiu K.H. (2022). Role of Cardiomyocyte-Derived Exosomal MicroRNA-146a-5p in Macrophage Polarization and Activation. Dis. Markers.

[74] Ibrahim A.G.E., Cheng K., Marbán E. (2014). Exosomes as critical agents of cardiac regeneration triggered by cell therapy. Stem Cell Rep..

[75] Liao Y., Li H., Cao H., Dong Y., Gao L., Liu Z., Ge J., Zhu H. (2021). Therapeutic silencing miR-146b-5p improves cardiac remodeling in a porcine model of myocardial infarction by modulating the wound reparative phenotype. Protein Cell.

[76] Kazimierczyk E., Eljaszewicz A., Zembko P., Tarasiuk E., Rusak M., Kulczynska-Przybik A., Lukaszewicz-Zajac M., Kaminski K., Mroczko B., Szmitkowski M. (2019). The relationships among monocyte subsets, miRNAs and inflammatory cytokines in patients with acute myocardial infarction. Pharmacol. Rep..

[77] Koga Y., Yasunaga M., Moriya Y., Akasu T., Fujita S., Yamamoto S., Matsumura Y. (2011). Exosome can Prevent RNase from Degrading MicroRNA in Feces. J. Gastrointest. Oncol..

[78] Zhou J., Chaudhry H., Zhong Y., Ali M.M., Perkin L.A., Owens W.B., Morales J.E., McGuire F.R., Zumbrun E.E., Zhang J. (2015). Dysregulation in microRNA Expression in Peripheral Blood Mononuclear Cells of Sepsis Patients is Associated with Immunopathology. Cytokine.

[79] Huang W., Tian S.S., Hang P.Z., Sun C., Guo J., Du Z.M. (2016). Combination of microRNA-21 and microRNA-146a Attenuates Cardiac Dysfunction and Apoptosis During Acute Myocardial Infarction in Mice. Mol. Ther. Nucleic Acids.

[80] Zhu Y., Zou C., Jia Y., Zhang H., Ma X., Zhang J. (2020). Knockdown of circular RNA circMAT2B reduces oxygen-glucose deprivation-induced inflammatory injury in H9c2 cells through up-regulating miR-133. Cell Cycle.

[81] Bian Y., Pang P., Li X., Yu S., Wang X., Liu K., Ju J., Wu H., Gao Y., Liu Q. (2021). CircHelz activates NLRP3 inflammasome to promote myocardial injury by sponging miR-133a-3p in mouse ischemic heart. J. Mol. Cell. Cardiol..

[82] Chu X., Wang Y., Pang L., Huang J., Sun X., Chen X. (2018). miR-130 aggravates acute myocardial infarction-induced myocardial injury by targeting PPAR-γ. J. Cell. Biochem..

[83] Xin H., Li C., Cai T., Cao J., Wang M. (2022). LncRNA KCNQ1OT1 contributes to hydrogen peroxide-induced apoptosis, inflammation, and oxidative stress of cardiomyocytes via miR-130a-3p/ZNF791 axis. Cell Biol. Int..

[84] Yang J., Xiang Z., Zhang J., Yang J., Zhai Y. (2021). miR-24 Alleviates MI/RI by Blocking the S100A8/TLR4/MyD88/NF-kB Pathway. J. Cardiovasc. Pharmacol..

[85] Lang B., Shang C., Meng L.R. (2016). Targeted silencing of S100A8 gene by miR-24 to increase chemotherapy sensitivity of endometrial carcinoma cells to paclitaxel. Med. Sci. Monit..

[86] Pruenster M., Vogl T., Roth J., Sperandio M. (2016). S100A8/A9: From basic science to clinical application. Pharmacol. Ther..

[87] Qiao S., Zhang W., Yin Y., Wei Z., Chen F., Zhao J., Sun X., Mu D., Xie J., Xu B. (2020). Extracellular vesicles derived from krüppel-Like factor 2-overexpressing endothelial cells attenuate myocardial ischemia-reperfusion injury by preventing Ly6Chigh monocyte recruitment. Theranostics.

[88] Qin D., Wang X., Li Y., Yang L., Wang R., Peng J., Mu K.E.X., Peng T., Han Q., Yu K.J. (2016). MicroRNA-223-5p and -3p Cooperatively suppress necroptosis in ischemic/reperfused hearts. J. Biol. Chem..

[89] Zhang L., Yang J., Guo M., Hao M. (2022). MiR-223-3p affects myocardial inflammation and apoptosis following myocardial infarction via targeting FBXW7. J. Thorac. Dis..

[90] Kuai X., Li L., Chen R., Wang K., Chen M., Cui B., Zhang Y., Li J., Zhu H., Zhou H. (2019). SCFFBXW7/GSK3β-mediated GFI1 degradation suppresses proliferation of gastric cancer cells. Cancer Res..

[91] Zhao Y., Wang C., Cui T., Wang Q., Xu Y., Miao C., Liu S. (2022). LncRNA FGD5-AS1 reduces cardiomyocyte apoptosis and inflammation by modulating Akt and miR-223-3p expression. Am. J. Transl. Res..

[92] Yao J., Shi Z., Ma X., Xu D., Ming G. (2019). lncRNA GAS5/miR-223/NAMPT axis modulates the cell proliferation and senescence of endothelial progenitor cells through PI3K/AKT signaling. J. Cell. Biochem..

[93] Kain V., Ingle K.A., Rajasekaran N.S., Halade G.V. (2021). Activation of EP4 receptor limits transition of acute to chronic heart failure in lipoxygenase deficient mice. Theranostics.

[94] Yang J., Fan Z., Yang J., Ding J., Yang C., Chen L. (2016). MicroRNA-22 attenuates myocardial ischemia-reperfusion injury via an anti-inflammatory mechanism in rats. Exp. Ther. Med..

[95] Forini F., Kusmic C., Nicolini G., Mariani L., Zucchi R., Matteucci M., Iervasi G., Pitto L. (2014). Triiodothyronine prevents cardiac ischemia/reperfusion mitochondrial impairment and cell loss by regulating miR30a/p53 axis. Endocrinology.

[96] Dhakshinamoorthy S., Jain A.K., Bloom D.A., Jaiswal A.K. (2005). Bach1 competes with Nrf2 leading to negative regulation of the antioxidant response element (ARE)-mediated NAD(P)H:quinone oxidoreductase 1 gene expression and induction in response to antioxidants. J. Biol. Chem.

[97] Sun M., Guo M., Ma G., Zhang N., Pan F., Fan X., Wang R. (2021). MicroRNA-30c-5p protects against myocardial ischemia/reperfusion injury via regulation of Bach1/Nrf2. Toxicol. Appl. Pharmacol..

[98] Chen J., Zhang M., Zhang S., Wu J., Xue S. (2020). Rno-microRNA-30c-5p promotes myocardial ischemia reperfusion injury in rats through activating NF-κB pathway and targeting SIRT1. BMC Cardiovasc. Disord..

[99] Wang M., Liu M., Ni T., Liu Q. (2018). miR-214 mediates vascular inflammation and apoptosis via PTEN expression. Mol. Med. Rep..

[100] Chen Y., Yin Y., Jiang H. (2021). miR-30e-5p Alleviates Inflammation and Cardiac Dysfunction After Myocardial Infarction Through Targeting PTEN. Inflammation.

[101] Zhu J., Yao K., Wang Q., Guo J., Shi H., Ma L., Liu H., Gao W., Zou Y., Ge J. (2016). Ischemic Postconditioning-Regulated miR-499 Protects the Rat Heart Against Ischemia/Reperfusion Injury by Inhibiting Apoptosis through PDCD4. Cell. Physiol. Biochem..

[102] Zhang X.Y., Huang Z., Li Q.J., Zhong G.Q., Meng J.J., Wang D.X., Tu R.H. (2020). Ischemic postconditioning attenuates the inflammatory response in ischemia/reperfusion myocardium by upregulating miR-499 and inhibiting TLR2 activation. Mol. Med. Rep..

[103] Ha T., Hu Y., Liu L., Lu C., McMullen J.R., Kelley J., Kao R.L., Williams D.L., Gao X., Li C. (2010). TLR2 ligands induce cardioprotection against ischaemia/reperfusion injury through a PI3K/Akt-dependent mechanism. Cardiovasc. Res..

[104] Wang Y., Ge P., Yang L., Wu C., Zha H., Luo T., Zhu Y. (2014). Protection of ischemic post conditioning against transient focal ischemia-induced brain damage is associated with inhibition of neuroinflammation via modulation of TLR2 and TLR4 pathways. J. Neuroinflammation.

[105] Zhou R., Huang W., Fan X., Liu F., Luo L., Yuan H., Jiang Y., Xiao H., Zhou Z., Deng C. (2019). miR-499 released during myocardial infarction causes endothelial injury by targeting α7-nAchR. J. Cell. Mol. Med..

[106] Wang N., Yu Y. (2022). MiR-486 alleviates hypoxia/reoxygenation-induced H9c2 cell injury by regulating forkhead box D3. Eur. Rev. Med. Pharmacol. Sci..

[107] Huang Y., Qi Y., Du J.Q., Zhang D.F. (2014). MicroRNA-34a regulates cardiac fibrosis after myocardial infarction by targeting Smad4. Expert Opin. Ther. Targets.

[108] Zhang C., Zhang Y., Zhu H., Hu J., Xie Z. (2018). MiR-34a/miR-93 target c-Ski to modulate the proliferaton of rat cardiac fibroblasts and extracellular matrix deposition in vivo and in vitro. Cell. Signal..

[109] Wang J., Zhang S., Li X., Gong M. (2020). LncRNA SNHG7 promotes cardiac remodeling by upregulating ROCK1 via sponging miR-34-5p. Aging.

[110] Wang Y.S., Li S.H., Guo J., Mihic A., Wu J., Sun L., Davis K., Weisel R.D., Li R.K. (2014). Role of miR-145 in cardiac myofibroblast differentiation. J. Mol. Cell. Cardiol..

[111] Song H.F., He S., Li S.H., Wu J., Yin W., Shao Z., Du G.-Q., Wu J., Li J., Weisel R.D. (2020). Knock-out of MicroRNA 145 impairs cardiac fibroblast function and wound healing post-myocardial infarction. J. Cell. Mol. Med..

[112] Cui S., Liu Z., Tao B., Fan S., Pu Y., Meng X., Li D., Xia H., Xu L. (2021). miR-145 attenuates cardiac fibrosis through the AKT/GSK-3β/β-catenin signaling pathway by directly targeting SOX9 in fibroblasts. J. Cell. Biochem..

[113] Huang S., Zhang L., Song J., Wang Z., Huang X., Guo Z., Chen F., Zhao X. (2019). Long noncoding RNA MALAT1 mediates cardiac fibrosis in experimental postinfarct myocardium mice model. J. Cell. Physiol..

[114] Vaskova E., Ikeda G., Tada Y., Wahlquist C., Mercola M., Yang P.C. (2020). Sacubitril/valsartan improves cardiac function and decreases myocardial fibrosis via downregulation of exosomal mir-181a in a rodent chronic myocardial infarction model. J. Am. Heart Assoc..

[115] Chen P., Pan J., Zhang X., Shi Z., Yang X. (2018). The role of microRNA-181a in myocardial fibrosis following myocardial infarction in a rat model. Med. Sci. Monit..

[116] Bruen R., Fitzsimons S., Belton O. (2019). MiR-155 in the resolution of atherosclerosis. Front. Pharmacol..

[117] Schumacher D., Curaj A., Simsekyilmaz S., Schober A., Liehn E.A., Mause S.F. (2021). Mir155 deficiency reduces myofibroblast density but fails to improve cardiac function after myocardial infarction in dyslipidemic mouse model. Int. J. Mol. Sci..

[118] Zhang S., Wang N., Ma Q., Fan F., Ma X. (2021). LncRNA TUG1 acts as a competing endogenous RNA to mediate CTGF expression by sponging miR-133b in myocardial fibrosis after myocardial infarction. Cell. Biol. Int..

[119] Arango D., Diosa-toro M., Rojas-hernandez L.S., Jessica L., Schwartz S.J., Mo X., Jiang J., Schmittgen T.D., Doseff A.I. (2015). Dietary apigenin reduces LPS-induced expression of miR-155 restoring immune balance during inflammation. Mol. Nutr. Food Res..

[120] Eissa M.G., Artlett C.M. (2019). The microRNA miR-155 is essential in fibrosis. Non-Coding RNA.

[121] Zhang H., Ma J., Liu F., Zhang J. (2021). Long non-coding RNA XIST promotes the proliferation of cardiac fibroblasts and the accumulation of extracellular matrix by sponging microRNA-155-5p. Exp. Ther. Med..

[122] Dakhlallah D., Zhang J., Yu L., Marsh C.B., Angelos M.G., Khan M. (2015). MicroRNA-133a engineered mesenchymal stem cells augment cardiac function and cell survival in the infarct heart. J. Cardiovasc. Pharmacol..

[123] Yu B.T., Yu N., Wang Y., Zhang H., Wan K., Sun X., Zhang C.S. (2019). Role of MIR-133a in regulating TGF-β1 signaling pathway in myocardial fibrosis after acute myocardial infarction in rats. Eur. Rev. Med. Pharmacol. Sci..

[124] Zhu W., Sun L., Zhao P., Liu Y., Zhang J., Zhang Y., Hong Y., Zhu Y., Lu Y., Zhao W. (2021). Macrophage migration inhibitory factor facilitates the therapeutic efficacy of mesenchymal stem cells derived exosomes in acute myocardial infarction through upregulating miR-133a-3p. J. Nanobiotechnology.

[125] Hong Y., Cao H., Wang Q., Ye J., Sui L., Feng J., Cai X., Song H., Zhang X., Chen X. (2016). MiR-22 may Suppress Fibrogenesis by Targeting TGFβR i in Cardiac Fibroblasts. Cell. Physiol. Biochem..

[126] Zhang L., Yin H., Jiao L., Liu T., Gao Y., Shao Y., Zhang Y., Shan H., Zhang Y., Yang B. (2018). Abnormal Downregulation of Caveolin-3 Mediates the Pro-Fibrotic Action of MicroRNA-22 in a Model of Myocardial Infarction. Cell. Physiol. Biochem..

[127] Zhao X.S., Ren Y., Wu Y., Ren H.K., Chen H. (2020). MiR-30b-5p and miR-22-3p restrain the fibrogenesis of post-myocardial infarction in mice via targeting PTAFR. Eur. Rev. Med. Pharmacol. Sci..

[128] Yuan J., Chen H., Ge D., Xu Y., Xu H., Yang Y., Gu M., Zhou Y., Zhu J., Ge T. (2017). Mir-21 Promotes Cardiac Fibrosis after Myocardial Infarction Via Targeting Smad7. Cell. Physiol. Biochem..

[129] Cao W., Shi P., Ge J.J. (2017). miR-21 enhances cardiac fibrotic remodeling and fibroblast proliferation via CADM1/STAT3 pathway. BMC Cardiovasc. Disord..

[130] Zhu L., Chen S., Chen Y. (2011). Unraveling the biological functions of Smad7 with mouse models. Cell Biosci..

[131] Dong X., Liu S., Zhang L., Yu S., Huo L., Qile M., Liu L., Yang B., Yu J. (2015). Downregulation of miR-21 is Involved in Direct Actions of Ursolic Acid on the Heart: Implications for Cardiac Fibrosis and Hypertrophy. Cardiovasc. Ther..

[132] Thum T., Gross C., Fiedler J., Fischer T., Kissler S., Bussen M., Galuppo P., Just S., Rottbauer W., Frantz S. (2008). MicroRNA-21 contributes to myocardial disease by stimulating MAP kinase signalling in fibroblasts. Nature.

[133] Zhang Y., Yuan B., Xu Y., Zhou N., Zhang R., Lu L., Feng Z. (2022). MiR-208b/miR-21 Promotes the Progression of Cardiac Fibrosis Through the Activation of the TGF-β1/Smad-3 Signaling Pathway: An in vitro and in vivo Study. Front. Cardiovasc. Med..

[134] Chiang M.H., Liang C.J., Lin L.C., Yang Y.F., Huang C.C., Chen Y.H., Kao H.L., Chen Y.C., Ke S.R., Lee C.W. (2020). miR-26a attenuates cardiac apoptosis and fibrosis by targeting ataxia–telangiectasia mutated in myocardial infarction. J. Cell. Physiol..

[135] Ge Z.W., Zhu X.L., Wang B.C., Hu J.L., Sun J.J., Wang S., Chen X.J., Meng S.P., Liu L., Cheng Z.Y. (2019). MicroRNA-26b relieves inflammatory response and myocardial remodeling of mice with myocardial infarction by suppression of MAPK pathway through binding to PTGS2. Int. J. Cardiol..

[136] Van Rooij E., Sutherland L.B., Thatcher J.E., DiMaio J.M., Naseem R.H., Marshall W.S., Hill J.A., Olson E.N. (2008). Dysregulation of microRNAs after myocardial infarction reveals a role of miR-29 in cardiac fibrosis. Proc. Natl. Acad. Sci. USA.

[137] Xue Y., Fan X., Yang R., Jiao Y., Li Y. (2020). miR-29b-3p inhibits post-infarct cardiac fibrosis by targeting FOS. Biosci. Rep..

[138] Zhang Y., Huang X.R., Wei L.H., Chung A.C., Yu C.M., Lan H.Y. (2014). MiR-29b as a therapeutic agent for angiotensin ii-induced cardiac fibrosis by targeting TGF-β/Smad3 signaling. Mol. Ther..

[139] Wang Y., Jin B.J., Chen Q., Yan B.J., Liu Z.L. (2019). MicroRNA-29b upregulation improves myocardial fibrosis and cardiac function in myocardial infarction rats through targeting SH2B3. Eur. Rev. Med. Pharmacol. Sci..

[140] Wang R., Peng L., Lv D., Shang F., Yan J., Li G., Li D., Ouyang J., Yang J. (2021). Leonurine Attenuates Myocardial Fibrosis Through Upregulation of miR-29a-3p in Mice Post-myocardial Infarction. J. Cardiovasc. Pharmacol..

[141] Yang F., Li P., Li H., Shi Q., Li S., Zhao L. (2015). MicroRNA-29b mediates the antifibrotic effect of tanshinone IIA in postinfarct cardiac remodeling. J. Cardiovasc. Pharmacol..

[142] Zhu J.N., Chen R., Fu Y.H., Lin Q.X., Huang S., Guo L.L., Zhang M.Z., Deng C.Y., Zou X., Zhong S.L. (2013). Smad3 Inactivation and MiR-29b Upregulation Mediate the Effect of Carvedilol on Attenuating the Acute Myocardium Infarction-Induced Myocardial Fibrosis in Rat. PLoS ONE.

[143] Zhou J., Zhou Y., Wang C.X. (2019). LncRNA-MIAT regulates fibrosis in hypertrophic cardiomyopathy (HCM) by mediating the expression of miR-29a-3p. J. Cell. Biochem..

[144] Chen L., Ji Q., Zhu H., Ren Y., Fan Z., Tian N. (2018). miR-30a attenuates cardiac fibrosis in rats with myocardial infarction by inhibiting CTGF. Exp. Ther. Med..

[145] Duisters R.F., Tijsen A.J., Schroen B., Leenders J.J., Lentink V., Van Der Made I., Herias V., Van Leeuwen R.E., Schellings M.W., Barenbrug P. (2009). MiR-133 and miR-30 Regulate connective tissue growth factor: Implications for a role of micrornas in myocardial matrix remodeling. Circ. Res..

[146] Li J., Salvador A.M., Li G., Valkov N., Ziegler O., Yeri A., Xiao C.Y., Meechoovet B., Alsop E., Rodosthenous R.S. (2021). Mir-30d Regulates Cardiac Remodeling by Intracellular and Paracrine Signaling. Circ. Res..

[147] Wang X., Yong C., Yu K., Yu R., Zhang R., Yu L., Li S., Cai S. (2018). Long noncoding RNA (lncRNA) n379519 promotes cardiac fibrosis in post-infarct myocardium by targeting mir-30. Med. Sci. Monit..

[148] Wang J., Huang W., Xu R., Nie Y., Cao X., Meng J., Xu X., Hu S., Zheng Z. (2012). MicroRNA-24 regulates cardiac fibrosis after myocardial infarction. J. Cell. Mol. Med..

[149] Tao L., Bei Y., Chen P., Lei Z., Fu S., Zhang H., Xu J., Che L., Chen X., Sluijter J.P.G. (2016). Crucial role of miR-433 in regulating cardiac fibrosis. Theranostics.

[150] Ye Q., Liu Q., Ma X., Bai S., Chen P., Zhao Y., Bai C., Liu Y., Liu K., Xin M. (2021). MicroRNA-146b-5p promotes atrial fibrosis in atrial fibrillation by repressing TIMP4. J. Cell. Mol. Med..

[151] Shafei S., Khanmohammadi M., Ghanbari H., Nooshabadi V.T., Tafti S.H.A., Rabbani S., Kasaiyan M., Basiri M., Tavoosidana G. (2022). Effectiveness of exosome mediated miR-126 and miR-146a delivery on cardiac tissue regeneration. Cell Tissue Res..

[152] Chen H., Lv L., Liang R., Guo W., Liao Z., Chen Y., Zhu K., Huang R., Zhao H., Pu Q. (2022). miR-486 improves fibrotic activity in myocardial infarction by targeting SRSF3/p21-Mediated cardiac myofibroblast senescence. J. Cell. Mol. Med..

[153] Barile L., Lionetti V., Cervio E., Matteucci M., Gherghiceanu M., Popescu L.M., Torre T., Siclari F., Moccetti T., Vassalli G. (2014). Extracellular vesicles fromhuman cardiac progenitor cells inhibit cardiomyocyte apoptosis and improve cardiac function aftermyocardial infarction. Cardiovasc. Res..

[154] Wang G., Wang R., Ruan Z., Liu L., Li Y., Zhu L. (2020). MicroRNA-132 attenuated cardiac fibrosis in myocardial infarction-induced heart failure rats. Biosci. Rep..

[155] Feng Y., Bao Y., Ding J., Li H., Liu W., Wang X., Guan H., Chen Z. (2022). MicroRNA-130a attenuates cardiac fibrosis after myocardial infarction through TGF-β/Smad signaling by directly targeting TGF-β receptor 1. Bioengineered.

[156] Morelli M.B., Shu J., Sardu C., Matarese A., Santulli G. (2020). Cardiosomal microRNAs are essential in post-infarction myofibroblast phenoconversion. Int. J. Mol. Sci..

[157] Yuan M., Zhang L., You F., Zhou J., Ma Y., Yang F., Tao L. (2017). MiR-145-5p regulates hypoxia-induced inflammatory response and apoptosis in cardiomyocytes by targeting CD40. Mol. Cell. Biochem..

[158] Sun N., Meng F., Xue N., Pang G., Wang Q., Ma H. (2018). Inducible miR-145 expression by HIF-1α protects cardiomyocytes against apoptosis via regulating SGK1 in simulated myocardial infarction hypoxic microenvironment. Cardiol. J..

[159] Xu H., Cao H., Zhu G., Liu S., Li H. (2017). Overexpression of microRNA-145 protects against rat myocardial infarction through targeting PDCD4. Am. J. Transl. Res..

[160] Tan L., Liu L., Yao J., Piao C. (2021). MiR-145-5p attenuates inflammatory response and apoptosis in myocardial ischemia-reperfusion injury by inhibiting (NADPH) oxidase homolog 1. Exp. Anim..

[161] Liang C., Wang S., Zhao L., Han Y., Zhang M. (2022). Effects of miR-145-5p on cardiomyocyte proliferation and apoptosis, GIGYF1 expression and oxidative stress response in rats with myocardial ischemia-reperfusion. Cell. Mol. Biol..

[162] Yang Y., Cheng H.W., Qiu Y., Dupee D., Noonan M., Lin Y.D., Fisch S., Unno K., Sereti K.I., Liao R. (2015). MicroRNA-34a Plays a Key Role in Cardiac Repair and Regeneration Following Myocardial Infarction. Circ. Res..

[163] Boon R.A., Iekushi K., Lechner S., Seeger T., Fischer A., Heydt S., Kaluza D., Tréguer K., Carmona G., Bonauer A. (2013). MicroRNA-34a regulates cardiac ageing and function. Nature.

[164] Kang H.J., Kang W.S., Hong M.H., Choe N., Kook H., Jeong H.C., Kang J., Hur J., Jeong M.H., Kim Y.S. (2015). Involvement of miR-34c in high glucose-insulted mesenchymal stem cells leads to inefficient therapeutic effect on myocardial infarction. Cell. Signal..

[165] Wang L., Li Q., Diao J., Lin L., Wei J. (2021). MiR-23a Is Involved in Myocardial Ischemia/Reperfusion Injury by Directly Targeting CX43 and Regulating Mitophagy. Inflammation.

[166] Song Y.S., Joo H.W., Park I.H., Shen G.Y., Lee Y., Shin J.H., Kim H., Kim K.S. (2017). Bone marrow mesenchymal stem cell-derived vascular endothelial growth factor attenuates cardiac apoptosis via regulation of cardiac miRNA-23a and miRNA-92a in a rat model of myocardial infarction. PLoS ONE.

[167] Luo Q., Guo D., Liu G., Chen G., Hang M., Jin M. (2018). Exosomes from MiR-126-Overexpressing Adscs Are Therapeutic in Relieving Acute Myocardial Ischaemic Injury. Cell. Physiol. Biochem..

[168] Xiao L., Gu Y., Ren G., Chen L., Liu L., Wang X., Gao L. (2021). MiRNA-146a Mimic Inhibits NOX4/P38 Signalling to Ameliorate Mouse Myocardial Ischaemia Reperfusion (I/R) Injury. Oxidative Med. Cell. Longev..

[169] Moghadam Z.M., Henneke P., Kolter J. (2021). From Flies to Men: ROS and the NADPH Oxidase in Phagocytes. Front. Cell Dev. Biol..

[170] Wang S., Ding L., Ji H., Xu Z., Liu Q., Zheng Y. (2016). The role of p38 MAPK in the development of diabetic cardiomyopathy. Int. J. Mol. Sci..

[171] Yang J., Yang J., Chen L., Ding J., Li S., Wu H., Zhang J., Fan Z., Dong W., Li X. (2014). MicroRNA-22 targeting CBP protects against myocardial ischemia-reperfusion injury through anti-apoptosis in rats. Mol. Biol. Rep..

[172] Wang Y., Chen J., Cowan D.B., Wang1 D.Z. (2021). Non-coding RNAs in cardiac regeneration: Mechanism of action and therapeutic potential. Semin. Cell Dev. Biol..

[173] Qiao L., Hu S., Liu S., Zhang H., Ma H., Huang K., Li Z., Su T., Vandergriff A., Tang J. (2019). MicroRNA-21-5p dysregulation in exosomes derived from heart failure patients impairs regenerative potential. J. Clin. Investig..

[174] Liao Z., Chen Y., Duan C., Zhu K., Huang R., Zhao H., Hintze M., Pu Q., Yuan Z., Lv L. (2020). Cardiac telocytes inhibit cardiac microvascular endothelial cell apoptosis through exosomal miRNA-21-5p-targeted cdip1 silencing to improve angiogenesis following myocardial infarction. Theranostics.

[175] Asangani I.A., Rasheed S.A.K., Nikolova D.A., Leupold J.H., Colburn N.H., Post S., Allgayer H. (2008). MicroRNA-21 (miR-21) post-transcriptionally downregulates tumor suppressor Pdcd4 and stimulates invasion, intravasation and metastasis in colorectal cancer. Oncogene.

[176] Lu Z., Liu M., Stribinskis V., Klinge C.M., Ramos K.S., Colburn N.H., Li Y. (2008). MicroRNA-21 promotes cell transformation by targeting the programmed cell death 4 gene. Oncogene.

[177] Zhou X.H., Chai H.X., Bai M., Zhang Z. (2020). LncRNA-GAS5 regulates PDCD4 expression and mediates myocardial infarction-induced cardiomyocytes apoptosis via targeting MiR-21. Cell Cycle.

[178] Zhang J.C., Xia L., Jiang Y., Wu D.Q., Liu S.C., Zhou X.N., Zhang F.X. (2019). Effect of lncRNA GAS5 on rats with acute myocardial infarction through regulating MIR-21. Eur. Rev. Med. Pharmacol. Sci..

[179] Meloni M., Marchetti M., Garner K., Littlejohns B., Sala-Newby G., Xenophontos N., Floris I., Suleiman M.S., Madeddu P., Caporali A. (2013). Local inhibition of microRNA-24 improves reparative angiogenesis and left ventricle remodeling and function in mice with myocardial infarction. Mol. Ther..

[180] Minghua W., Zhijian G., Chahua H., Qiang L., Minxuan X., Luqiao W., Weifang Z., Peng L., Biming Z., Lingling Y. (2018). Plasma exosomes induced by remote ischaemic preconditioning attenuate myocardial ischaemia/reperfusion injury by transferring MIR-24 article. Cell Death Dis..

[181] Mohammadi A., Karami A.R.B., Mashtani V.D., Sahraei T., Tarashoki Z.B., Khattavian E., Mobarak S., Kazerouni H.M., Radmanesh E. (2021). Evaluation of Oxidative Stress, Apoptosis, and Expression of MicroRNA-208a and MicroRNA-1 in Cardiovascular Patients. Rep. Biochem. Mol. Biol..

[182] Shyu K.G., Wang B.W., Cheng W.P., Lo H.M. (2015). MicroRNA-208a increases myocardial endoglin expression and myocardial fibrosis in acute myocardial infarction. Can. J. Cardiol..

[183] Lesizza P., Prosdocimo G., Martinelli V., Sinagra G., Zacchigna S., Giacca M. (2017). Single-Dose Intracardiac Injection of Pro-Regenerative MicroRNAs Improves Cardiac Function after Myocardial Infarction. Circ. Res..

[184] Lock M.C., Tellam R.L., Botting K.J., Wang K.C.W., Selvanayagam J.B., Brooks D.A., Seed M., Morrison J.L. (2018). The role of miRNA regulation in fetal cardiomyocytes, cardiac maturation and the risk of heart disease in adults. J. Physiol..

[185] Porrello E.R., Johnson B.A., Aurora A.B., Simpson E., Nam Y.-J., Matkovich S.J., Dornall G.W., van Rooij E., Olson E.N. (2011). miR-15 family regulates CM proliferation. Circ. Res..

[186] Mathiyalagan P., Liang Y., Kim D., Misener S., Thorne T., Kamide C.E., Klyachko E., Losordo D.W., Hajjar R.J., Sahoo S. (2017). Angiogenic Mechanisms of Human CD34+ Stem Cell Exosomes in the Repair of Ischemic Hindlimb. Circ. Res..

[187] Mocharla P., Briand S., Giannotti G., Dörries C., Jakob P., Paneni F., Lüscher T., Landmesser U. (2013). AngiomiR-126 expression and secretion from circulating CD34+ and CD14+ PBMCs: Role for proangiogenic effects and alterations in type 2 diabetics. Blood.

[188] Zhang L., Wei Q., Liu X., Zhang T., Wang S., Zhou L., Zou L., Fan F., Chi H., Sun J. (2021). Exosomal microRNA-98-5p from hypoxic bone marrow mesenchymal stem cells inhibits myocardial ischemia–reperfusion injury by reducing TLR4 and activating the PI3K/Akt signaling pathway. Int. Immunopharmacol..

[189] Gupta S.K., Foinquinos A., Thum S., Remke J., Zimmer K., Bauters C., de Groote P., Boon R.A., de Windt L.J., Preissl S. (2016). Preclinical Development of a MicroRNA-Based Therapy for Elderly Patients With Myocardial Infarction. J. Am. Coll. Cardiol..

[190] Zhu W., Yang L., Shan H., Zhang Y., Zhou R., Su Z., Du Z. (2011). Microrna expression analysis: Clinical advantage of propranolol reveals key micrornas in myocardial infarction. PLoS ONE.

[191] Zhou J., He S., Wang B., Yang W., Zheng Y., Jiang S., Li D., Lin J. (2022). Construction and Bioinformatics Analysis of circRNA-miRNA-mRNA Network in Acute Myocardial Infarction. Front. Genet..

[192] Ma R., Gao L., Liu Y., Du P., Chen X., Li G. (2021). LncRNA TTTY15 knockdown alleviates H2O2-stimulated myocardial cell injury by regulating the miR-98-5p/CRP pathway. Mol. Cell. Biochem..

[193] Chen B., Luo L., Wei X., Gong D., Li Z., Li S., Tang W., Jin L. (2021). M1 Bone Marrow-Derived Macrophage-Derived Extracellular Vesicles Inhibit Angiogenesis and Myocardial Regeneration following Myocardial Infarction via the MALAT1/MicroRNA-25-3p/CDC42 Axis. Oxidative Med. Cell. Longev..

[194] Chen Z.L., Chen Y.X., Zhou J., Li Y., Gong C.Y., Wang X.B. (2020). LncRNA HULC alleviates HUVEC inflammation and improves angiogenesis after myocardial infarction through down-regulating miR-29b. Eur. Rev. Med. Pharmacol. Sci..

[195] Liang H., Li F., Li H., Wang R., Du M. (2020). Overexpression of lncRNA HULC Attenuates Myocardial Ischemia/reperfusion Injury in Rat Models and Apoptosis of Hypoxia/reoxygenation Cardiomyocytes via Targeting miR-377-5p through NLRP3/Caspase-1/IL-1β Signaling Pathway Inhibition. Immunol. Investig..

[196] Zhang H., Zhao J., Shao P. (2020). Long noncoding RNA MIAT2 alleviates lipopolysaccharide-induced inflammatory damage in WI-38 cells by sponging microRNA-15. J. Cell. Physiol..

[197] Xin M., Liang H., Wang H., Wen D., Wang L., Zhao L., Sun M., Wang J. (2019). Mirt2 functions in synergy with miR-377 to participate in inflammatory pathophysiology of Sjögren’s syndrome. Artif. Cells Nanomed. Biotechnol..

[198] Li T., Tian H., Li J., Zuo A., Chen J., Xu D., Guo Y., Gao H. (2019). Overexpression of lncRNA Gm2691 attenuates apoptosis and inflammatory response after myocardial infarction through PI3K/Akt signaling pathway. IUBMB Life.

[199] Li X., Zhou J., Huang K. (2017). Erratum: Inhibition of the lnc RNA Mirt1 attenuates acute myocardial infarction by suppressing NF-κB activation. Cell. Physiol. Biochem..

[200] Gast M., Rauch B.H., Haghikia A., Nakagawa S., Haas J., Stroux A., Schmidt D., Schumann P., Weiss S., Jensen L. (2019). Long noncoding RNA NEAT1 modulates immune cell functions and is suppressed in early onset myocardial infarction patients. Cardiovasc. Res..

[201] Lu W., Sheng Z., Zhang Z., Ma G., Chen L., Huang J., Ding J., Dai Q. (2020). LncRNA-LUNAR1 Levels Are Closely Related to Coronary Collaterals in Patients with Chronic Total Coronary Occlusion. J. Cardiovasc. Transl. Res..

[202] Zhang Y., Bian Y. (2020). Long Non-Coding RNA SNHG8 Plays a Key Role in Myocardial Infarction Through Affecting Hypoxia-Induced Cardiomyocyte Injury. Med. Sci. Monit..

[203] Hao K., Lei W., Wu H., Wu J., Yang Z., Yan S., Lu X.A., Li J., Xia X., Han X. (2019). LncRNA-Safe contributes to cardiac fibrosis through Safe-Sfrp2-HuR complex in mouse myocardial infarction. Theranostics.

[204] Micheletti R., Plaisance I., Abraham B.J., Sarre A., Ting C.-C., Alexanian M., Maric D., Maison D., Nemir M., Young R.A. (2017). The long noncoding RNA Wisper controls cardiac fibrosis and remodeling. Sci. Transl. Med..

[205] Luo S., Zhang M., Wu H., Ding X., Li D., Dong X., Hu X., Su S., Shang W., Wu J. (2021). SAIL: A new conserved anti-fibrotic lncRNA in the heart. Basic Res. Cardiol..

[206] Zhang F., Fu X., Kataoka M., Liu N., Wang Y., Gao F., Liang T., Dong X., Pei J., Hu X. (2021). Long noncoding RNA Cfast regulates cardiac fibrosis. Mol. Ther. Nucleic Acids.

[207] Sun F., Zhuang Y., Zhu H., Wu H., Li D., Zhan L., Yang W., Yuan Y., Xie Y., Yang S. (2019). LncRNA PCFL promotes cardiac fibrosis via miR-378/GRB2 pathway following myocardial infarction. J. Mol. Cell. Cardiol..

[208] Xiong X., Liu J., He Q., Dai R., Zhang H., Cao Z., Liao Y., Liu B., Zhou Y., Chen J. (2021). Long non-coding RNA NORAD aggravates acute myocardial infarction by promoting fibrosis and apoptosis via miR-577/COBLL1 axis. Environ. Toxicol..

[209] Liang H., Pan Z., Zhao X., Liu L., Sun J., Su X., Xu C., Zhou Y., Zhao D., Xu B. (2018). Erratum: LncRNA PFL contributes to cardiac fibrosis by acting as a competing endogenous RNA of let-7d. Theranostics.

[210] Lang M., Ou D., Liu Z., Li Y., Zhang X., Zhang F. (2021). LncRNA MHRT promotes cardiac fibrosis via miR-3185 pathway following myocardial infarction. Int. Heart J..

[211] Luo B., He Z., Huang S., Wang J., Han D., Xue H., Liu P., Zeng X., Lu D. (2020). Long Non-Coding RNA 554 Promotes Cardiac Fibrosis via TGF-β1 Pathway in Mice Following Myocardial Infarction. Front. Pharmacol..

[212] Chen G., Huang S., Song F., Zhou Y., He X. (2020). Lnc-Ang362 is a pro-fibrotic long non-coding RNA promoting cardiac fibrosis after myocardial infarction by suppressing Smad7. Arch. Biochem. Biophys..

[213] Zhuang Y., Li T., Zhuang Y., Li Z., Yang W., Huang Q., Li D., Wu H., Zhang G., Yang T. (2019). Involvement of lncR-30245 in Myocardial Infarction–Induced Cardiac Fibrosis Through Peroxisome Proliferator-Activated Receptor-γ–Mediated Connective Tissue Growth Factor Signalling Pathway. Can. J. Cardiol..

[214] Du L., Chen J., Wu Y., Xia G., Chen M., Zhao P., Wang Y., Yao D., Liu F., Zhang L. (2021). Long Non-coding RNA N1LR Protects Against Myocardial Ischemic/Reperfusion Injury Through Regulating the TGF-β Signaling Pathway. Front. Cardiovasc. Med..

[215] Liu Y., Wang T., Zhang M., Chen P., Yu Y. (2019). Down-regulation of myocardial infarction associated transcript 1 improves myocardial ischemia-reperfusion injury in aged diabetic rats by inhibition of activation of NF-κB signaling pathway. Chem. Interactions.

[216] Yan M., Liu Q., Jiang Y., Wang B., Ji Y., Liu H., Xie Y. (2020). Long Noncoding RNA LNC_000898 Alleviates Cardiomyocyte Apoptosis and Promotes Cardiac Repair after Myocardial Infarction through Modulating the miR-375/PDK1 Axis. J. Cardiovasc. Pharmacol..

[217] Chen Y., Li S., Zhang Y., Wang M., Li X., Liu S., Xu D., Bao Y., Jia P., Wu N. (2021). The lncRNA Malat1 regulates microvascular function after myocardial infarction in mice via miR-26b-5p/Mfn1 axis-mediated mitochondrial dynamics. Redox Biol..

[218] Li L., Wang Q., Yuan Z., Chen A., Liu Z., Wang Z., Li H. (2018). LncRNA-MALAT1 promotes CPC proliferation and migration in hypoxia by up-regulation of JMJD6 via sponging miR-125. Biochem. Biophys. Res. Commun..

[219] Zhang B.F., Jiang H., Chen J., Hu Q., Yang S., Liu X.P., Liu G. (2020). LncRNA H19 ameliorates myocardial infarction-induced myocardial injury and maladaptive cardiac remodelling by regulating KDM3A. J. Cell. Mol. Med..

[220] Li L., Wang Q., Yuan Z., Chen A., Liu Z., Li H., Wang Z. (2018). Long non-coding RNA H19 contributes to hypoxia-induced CPC injury by suppressing Sirt1 through miR-200a-3p. Acta Biochim. Biophys. Sin..

[221] Luo H., Wang J., Liu D., Zang S., Ma N., Zhao L., Zhang L., Zhang X., Qiao C. (2018). The lncRNA H19/miR-675 axis regulates myocardial ischemic and reperfusion injury by targeting PPARα. Mol. Immunol..

[222] Choong O.K., Chen C.Y., Zhang J., Lin J.H., Lin P.J., Ruan S.C., Kamp T.J., Hsieh P.C.H. (2019). Hypoxia-induced H19/YB-1 cascade modulates cardiac remodeling after infarction. Theranostics.

[223] Zhao X., Ren Y., Ren H., Wu Y., Liu X., Chen H., Ying C. (2021). The mechanism of myocardial fibrosis is ameliorated by myocardial infarction-associated transcript through the PI3K/Akt signaling pathway to relieve heart failure. J. Int. Med. Res..

[224] Dong Q., Wang Q., Yan X., Wang X., Li Z., Zhang L. (2021). Long noncoding RNA MIAT inhibits the progression of diabetic nephropathy and the activation of NF-κB pathway in high glucose-treated renal tubular epithelial cells by the miR-182-5p/GPRC5A axis. Open Med..

[225] Sun Q., Luo M., Gao Z., Han X., Yan Z., Xie S., Zhao H., Sun H. (2021). TUG1 knockdown suppresses cardiac fibrosis after myocardial infarction. Mamm. Genome.

[226] Li M., Zheng H., Han Y., Chen Y., Li B., Chen G., Chen X., Huang S., He X., Wei G. (2021). LncRNA Snhg1-driven self-reinforcing regulatory network promoted cardiac regeneration and repair after myocardial infarction. Theranostics.

[227] Cai B., Ma W., Wang X., Sukhareva N., Hua B., Zhang L., Xu J., Li X., Li S., Liu S. (2020). Targeting LncDACH1 promotes cardiac repair and regeneration after myocardium infarction. Cell Death Differ..

[228] Fu W., Ren H., Shou J., Liao Q., Li L., Shi Y., Jose P.A., Zeng C., Wang W.E. (2022). Loss of NPPA-AS1 promotes heart regeneration by stabilizing SFPQ–NONO heteromer-induced DNA repair. Basic Res. Cardiol..

[229] Ponnusamy M., Liu F., Zhang Y.H., Li R.B., Zhai M., Liu F., Zhou L.Y., Liu C.Y., Yan K.W., Dong Y.H. (2019). Long Noncoding RNA CPR (Cardiomyocyte Proliferation Regulator) Regulates Cardiomyocyte Proliferation and Cardiac Repair. Circulation.

[230] Trembinski D.J., Bink D.I., Theodorou K., Sommer J., Fischer A., van Bergen A., Kuo C.C., Costa I.G., Schürmann C., Leisegang M.S. (2020). Aging-regulated anti-apoptotic long non-coding RNA Sarrah augments recovery from acute myocardial infarction. Nat. Commun..

[231] Hosen M.R., Militello G., Weirick T., Ponomareva Y., Dassanayaka S., MooreIV J.B., Döring C., Wysoczynski M., Jones S.P., Dimmeler S. (2018). Airn Regulates Igf2bp2 Translation in Cardiomyocytes. Circ. Res..

[232] Cai B., Ma W., Ding F., Zhang L., Huang Q., Wang X., Hua B., Xu J., Li J., Bi C. (2018). The Long Noncoding RNA CAREL Controls Cardiac Regeneration. J. Am. Coll. Cardiol..

[233] Li X., Sun Y., Huang S., Chen Y., Chen X., Li M., Si X., He X., Zheng H., Zhong L. (2019). Inhibition of AZIN2-sv induces neovascularization and improves prognosis after myocardial infarction by blocking ubiquitin-dependent talin1 degradation and activating the Akt pathway. EBioMedicine.

[234] Li X., He X., Wang H., Li M., Huang S., Chen G., Jing Y., Wang S., Chen Y., Liao W. (2018). Loss of AZIN2 splice variant facilitates endogenous cardiac regeneration. Cardiovasc. Res..

[235] Safaei S., Tahmasebi-Birgani M., Bijanzadeh M., Seyedian S.M. (2020). Increased expression level of long noncoding RNA H19 in plasma of patients with myocardial infarction. Int. J. Mol. Cell. Med..

[236] Yang L., Deng J., Ma W., Qiao A., Xu S., Yu Y., Boriboun C., Kang X., Han D., Ernst P. (2021). Ablation of lncrna miat attenuates pathological hypertrophy and heart failure. Theranostics.

[237] Zhu J., Chen Z., Peng X., Zheng Z., Le A., Guo J., Ma L., Shi H., Yao K., Zhang S. (2022). Extracellular Vesicle-Derived circITGB1 Regulates Dendritic Cell Maturation and Cardiac Inflammation via miR-342-3p/NFAM1. Oxidative Med. Cell. Longev..

[238] Ren K., Li B., Jiang L., Liu Z., Wu F., Zhang Y., Liu J., Duan W. (2021). Circ_0023461 Silencing Protects Cardiomyocytes from Hypoxia-Induced Dysfunction through Targeting miR-370-3p/PDE4D Signaling. Oxidative Med. Cell. Longev..

[239] Chaorui X., Jia Z., Cao X., Wang S., Wang J., An L. (2022). Hsa_circ_0007059 promotes apoptosis and inflammation in cardiomyocytes during ischemia by targeting microRNA-378 and microRNA-383. Cell Cycle.

[240] Zhang Y., Li Z., Wang J., Chen H., He R., Wu H. (2022). CircTRRAP Knockdown Has Cardioprotective Function in Cardiomyocytes via the Signal Regulation of miR-370-3p/PAWR Axis. Cardiovasc. Ther..

[241] Wang S., Cheng Z., Chen X., Lu G., Zhu X., Xu G. (2021). CircUBXN7 mitigates H/R-induced cell apoptosis and inflammatory response through the miR-622-MCL1 axis. Am. J. Transl. Res..

[242] Zhou D., Dai Z., Ren M., Yang M. (2022). Adipose-Derived Stem Cells-Derived Exosomes with High Amounts of Circ_0001747 Alleviate Hypoxia/Reoxygenation-Induced Injury in Myocardial Cells by Targeting MiR-199b-3p/MCL1 Axis. Int. Heart J..

[243] Li F., Long T.Y., Bi S.S., Sheikh S.A., Zhang C.L. (2020). circPAN3 exerts a profibrotic role via sponging miR-221 through FoxO3/ATG7-activated autophagy in a rat model of myocardial infarction. Life Sci..

[244] Sun L.Y., Zhao J.C., Ge X.M., Zhang H., Wang C.M., Bie Z.D. (2020). Circ_LAS1L regulates cardiac fibroblast activation, growth, and migration through miR-125b/SFRP5 pathway. Cell Biochem. Funct..

[245] Zhu Y., Pan W., Yang T., Meng X., Jiang Z., Tao L., Wang L. (2019). Upregulation of circular RNA CircNFIB attenuates cardiac fibrosis by sponging miR-433. Front. Genet..

[246] Wu N., Li C., Xu B., Xiang Y., Jia X., Yuan Z., Wu L., Zhong L., Li Y. (2021). Circular RNA mmu_circ_0005019 inhibits fibrosis of cardiac fibroblasts and reverses electrical remodeling of cardiomyocytes. BMC Cardiovasc. Disord..

[247] Li X.X., Mu B., Li X., Bie Z.D. (2022). circCELF1 Inhibits Myocardial Fibrosis by Regulating the Expression of DKK2 Through FTO/m6A and miR-636. J. Cardiovasc. Transl. Res..

[248] Wang Y., Li C., Zhao R., Qiu Z., Shen C., Wang Z., Liu W., Zhang W., Ge J., Shi B. (2021). CircUbe3a from M2 macrophage-derived small extracellular vesicles mediates myocardial fibrosis after acute myocardial infarction. Theranostics.

[249] Sun G., Shen J.F., Wei X.F., Qi G.X. (2021). Circular RNA foxo3 relieves myocardial ischemia/reperfusion injury by suppressing autophagy via inhibiting HMGB1 by repressing KAT7 in myocardial infarction. J. Inflamm. Res..

[250] Lan Z., Wang T., Zhang L., Jiang Z., Zou X. (2022). CircSLC8A1 Exacerbates Hypoxia-Induced Myocardial Injury via Interacting with MiR-214-5p to Upregulate TEAD1 Expression. Int. Heart J..

[251] Huang S., Li X., Zheng H., Si X., Li B., Wei G., Li C., Chen Y., Chen Y., Liao W. (2019). Loss of Super-Enhancer-Regulated circRNA Nfix Induces Cardiac Regeneration after Myocardial Infarction in Adult Mice. Circulation.

[252] Zhang M., Wang Z., Cheng Q., Wang Z., Lv X., Wang Z., Li N. (2020). Circular RNA (circRNA) CDYL induces myocardial regeneration by ceRNA after myocardial infarction. Med. Sci. Monit..

[253] Ma W., Wang X., Sun H., Xu B., Song R., Tian Y., Zhao L., Xu Y., Zhao Y., Yang F. (2022). Oxidant stress-sensitive circRNA Mdc1 controls cardiomyocyte chromosome stability and cell cycle re-entry during heart regeneration. Pharmacol. Res..

[254] Zheng H., Huang S., Wei G., Sun Y., Li C., Si X., Chen Y., Tang Z., Li X., Chen Y. (2022). CircRNA Samd4 induces cardiac repair after myocardial infarction by blocking mitochondria-derived ROS output. Mol. Ther..

[255] Hu X., Ma R., Cao J., Du X., Cai X., Fan Y. (2022). CircSAMD4A aggravates H/R-induced cardiomyocyte apoptosis and inflammatory response by sponging miR-138-5p. J. Cell. Mol. Med..

[256] Si X., Zheng H., Wei G., Li M., Li W., Wang H., Guo H., Sun J., Li C., Zhong S. (2020). CircRNA Hipk3 Induces Cardiac Regeneration after Myocardial Infarction in Mice by Binding to Notch1 and miR-133a. Mol. Ther. Nucleic Acids.

[257] Deng Y., Wang J., Xie G., Zeng X., Li H. (2019). Circ-hipk3 strengthens the effects of adrenaline in heart failure by mir-17-3p-adcy6 axis. Int. J. Biol. Sci..

[258] Bai M., Pan C.L., Jiang G.X., Zhang Y.M. (2020). CircRNA 010567 improves myocardial infarction rats through inhibiting TGF-β1. Eur. Rev. Med. Pharmacol. Sci..

[259] Groenewegen A., Rutten F.H., Mosterd A., Hoes A.W. (2020). Epidemiology of heart failure. Eur. J. Heart Fail..

[260] Truby L.K., Rogers J.G. (2020). Advanced Heart Failure: Epidemiology, Diagnosis, and Therapeutic Approaches. JACC Heart Fail..

[261] Dalen J.E., Alpert J.S., Goldberg R.J., Weinstein R.S. (2014). The epidemic of the 20th century: Coronary heart disease. Am. J. Med..

[262] Duggan J.P., Peters A.S., Trachiotis G.D., Antevil J.L. (2022). Epidemiology of Coronary Artery Disease. Surg. Clin. North Am..

[263] Poss K.D., Wilson L.G., Keating M.T. (2002). Heart regeneration in zebrafish. Science.

[264] Trajano L.F., Smart N. (2021). Immunomodulation for optimal cardiac regeneration: Insights from comparative analyses. NPJ Regen. Med..

